# Scholarly Context Not Found: One in Five Articles Suffers from Reference Rot

**DOI:** 10.1371/journal.pone.0115253

**Published:** 2014-12-26

**Authors:** Martin Klein, Herbert Van de Sompel, Robert Sanderson, Harihar Shankar, Lyudmila Balakireva, Ke Zhou, Richard Tobin

**Affiliations:** 1 Digital Library Research and Prototyping Team, Research Library, Los Alamos National Laboratory, Los Alamos, New Mexico, United States of America; 2 Language Technology Group, The University of Edinburgh, Edinburgh, Scotland, United Kingdom; Bar-Ilan University, Israel

## Abstract

The emergence of the web has fundamentally affected most aspects of information communication, including scholarly communication. The immediacy that characterizes publishing information to the web, as well as accessing it, allows for a dramatic increase in the speed of dissemination of scholarly knowledge. But, the transition from a paper-based to a web-based scholarly communication system also poses challenges. In this paper, we focus on reference rot, the combination of link rot and content drift to which references to web resources included in Science, Technology, and Medicine (STM) articles are subject. We investigate the extent to which reference rot impacts the ability to revisit the web context that surrounds STM articles some time after their publication. We do so on the basis of a vast collection of articles from three corpora that span publication years 1997 to 2012. For over one million references to web resources extracted from over 3.5 million articles, we determine whether the HTTP URI is still responsive on the live web and whether web archives contain an archived snapshot representative of the state the referenced resource had at the time it was referenced. We observe that the fraction of articles containing references to web resources is growing steadily over time. We find one out of five STM articles suffering from reference rot, meaning it is impossible to revisit the web context that surrounds them some time after their publication. When only considering STM articles that contain references to web resources, this fraction increases to seven out of ten. We suggest that, in order to safeguard the long-term integrity of the web-based scholarly record, robust solutions to combat the reference rot problem are required. In conclusion, we provide a brief insight into the directions that are explored with this regard in the context of the Hiberlink project.

## Introduction

### Reference Rot in Web-Based Scholarly Communication

Referencing sources is a fundamental part of the scholarly discourse. There is an expectation that referenced sources can and should be checked by others, to allow a correct interpretation of information that is being communicated and to support reproducibility of results. This credo continues as scholarly communication transitions from being a paper-based to a web-based endeavor. But, as research communication, and increasingly also the research process, transition to the web, the range of scholarly assets that are being communicated and referenced is greatly increasing. In the paper-based era, a scholarly article would typically reference other scholarly articles. In the web-based era, the scope of referencing crucially still includes articles but has extended to also cover a wide range of assets that are used or created during the research process such as software, ontologies, scientific workflows, datasets, online debates, presentations, blogs, videos, etc. Also, whereas references in the paper-based era were purely textual, in the web era they additionally include HTTP URIs - from here on referred to as URIs - that provide convenient and immediate access to referenced resources on the web. This immediacy is one of the web's transformative characteristics that is inherited by web-based scholarly communication and that allows for a dramatic increase in the speed of knowledge dissemination. But web-based scholarly communication also inherits some of the rather frustrating characteristics of the web, and, in this paper, we focus on one: reference rot.

**Reference rot** is a term we introduced in the Hiberlink project [1] to denote the combination of two problems involved in using URI references, both of which relate to the dynamic and ephemeral nature of the web:

- **Link rot**: The resource identified by a URI may cease to exist and hence a URI reference to that resource will no longer provide access to referenced content.
- **Content drift**: The resource identified by a URI may change over time and hence, the content at the end of the URI may evolve, even to such an extent that it ceases to be representative of the content that was originally referenced.

Link rot is known to all web users as they are regularly confronted with unhelpful “404 Not Found” error messages. Content drift, while very real, features less prominently as an annoyance of the web, probably because it rarely manifests itself during a browsing session but rather over an extended period of time. Both link rot and content drift pose a threat to the long-term persistence and integrity of the new-era scholarly record. Indeed, as references rot or as the content they originally referred to changes, it becomes impossible to revisit the intellectual context that surrounded the referencing article at the time of its publication. This is a significant step backwards when compared to the paper-based journal era: revisiting the context in those days was always possible even though it may have required visiting several libraries, each one a hub in a global, distributed archive for the journal literature.

This threat was recognized early on when journal articles found their way onto the web. In order to combat link rot, the Digital Object Identifier (DOI) was introduced to persistently identify journal articles. In addition, the DOI resolver [2] for the URI version of DOIs was introduced to ensure that web links pointing at these articles remain actionable, even when the articles change web location. A similar approach has meanwhile emerged for research data, which is increasingly regarded as an integral part of the scholarly record. Content drift is hardly a matter of concern for references to journal articles, because of the inherent fixity that, especially PDF-formated, articles exhibit. Nevertheless, special-purpose solutions for long-term digital archiving of the digital journal literature, such as LOCKSS [3], CLOCKSS [4], and Portico [5], have emerged to ensure that articles and the articles they reference can be revisited even if the portals that host them vanish from the web. More recently, the Keepers Registry [6] has been introduced to keep track of the extent to which the digital journal literature is archived by what memory organizations. These combined efforts ensure that it is possible to revisit the scholarly context that consists of articles referenced by a certain article long after its publication.

While solutions have been put in place to combat reference rot for references to journal articles, the problem has so far not been adequately addressed for references to **web at large resources** that are increasingly used in journal articles and that point to a wide range of web content, distinct from journal articles. In many cases, these web at large resources are regarded as supporting materials for a journal article, but accessing them can be essential for a comprehensive understanding of the described research. In other cases, such as in computational sciences, these resources may be the core result of research and an article is merely an advertisement for it [7]. It is not uncommon for communities on the web that are not overly concerned with persistence to be custodians of web resources that are referenced from scholarly articles including various types of web portals, scholarly wikis, or project and personal web sites with a limited lifespan. Lacking the consolidated preservation attention that journal articles receive, these materials run a significant risk of vanishing from the web. From prior research, we know they effectively do, and, as a consequence, links to these resources rot. In addition, many of these web at large resources lack the sense of fixity that journal articles typically have. Software, ontologies, scientific workflows are frequently updated as research efforts evolve, blogs attract comments over time, and project web sites evolve during the project lifespan. These resources, rather than being frozen in time, are subject to content drift. When revisiting a reference made to such a resource some time after it was referenced in a scholarly article, there is a significant chance that the content has evolved. With both link rot and content drift at work, and lacking special purpose solutions to combat them, it is unclear to which extent the scholarly context referenced by any given article can be revisited some time after its publication.

The research reported in this paper is aimed at assessing the extent to which the web at large context that surrounds journal articles can be revisited some time after their publication. It is the first result of the research track of the Hiberlink project that explores the impact that reference rot has on the scholarly literature. In order to answer our research question, we assembled three vast corpora consisting of more than 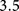 million scholarly articles spanning publication years 1997 to 2012. All articles were processed and over 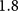 million were found to contain URI references. The total amount of URI references extracted from these papers amounted to almost 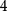 million, over 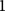 million being URI references to web at large resources that became the focus of our research. We set out to answer our research question using a multi-pronged approach that can be summarized as follows:

- Assessment of the extent to which the collection of URI references to web at large resources is subject to reference rot: The full web at large context that surrounds an article some time after its publication consists of two distinct components. The first component, the **current context**, entails the referenced resources as they exist on the live web. Revisiting this context requires the URI references to be active, not rotten, on the live web at the time one wants to revisit them. Hence, we set out to assess the extent of link rot for our collection of URI references. This part of our research follows the path paved by prior link rot studies but stands out due to the scale of the operation: the size of our source corpora and of our collection of URI references exceeds those of prior link rot studies by at least two and in most cases three orders of magnitude. The second component, the **past context**, entails the content that was available from the referenced resources at the time they were referenced. Based on the understanding - provided by research literature - that web resources change over time, this originally referenced content can not be revisited by following URI references on the live web. Rather, a snapshot of that content created around the time of referencing is required. If such a snapshot can be retrieved anywhere, it must be in one of the various web archives around the world. Hence, we consulted web archives to assess to which extent they contain archived snapshots - henceforth also named Mementos - that are representative for the content that was originally referenced. This part of our research has similarities with prior efforts that have studied the archival status of referenced URIs. However, our work involves polling multiple web archives, not just one, and operates at a vast scale. More importantly, prior studies merely checked whether an archival snapshot of a referenced URI was available in a web archive. In doing so, they ignored that - again as a result of content drift - such a snapshot needs to be taken close to the time a URI is being referenced in order for it to be representative of the originally referenced content. Our work is novel in that it introduces this notion of temporal representativeness of Mementos and assesses archival status, and hence the ability to revisit the originally referenced content, on this basis.
- Assessment of the loss of context: We consider the network that consists of articles and the web at large resources referenced by those articles. We provide an insight into the current context and the past context of this network, by showing how the referenced URIs are affected by link rot and by the lack of temporally representative Mementos, respectively.
- Assessment of the extent to which the three corpora are subject to reference rot: We introduce a typology that categorizes articles as **immune**, **healthy**, or **infected** by reference rot whereby infected indicates the inability to revisit the web at large context - current and past - that surrounds an article. For each of our corpora, we assess the distribution of articles according to this categorization.
- Assessment of the extent to which the STM literature at large is subject to reference rot: We use the same reference rot typology, and extrapolate the assessment obtained for one of our corpora to obtain an insight into the extent of reference rot in the STM literature.

To the best of our knowledge, neither the latter three approaches nor the notion of temporal representativeness of Mementos have been explored by prior studies.

### Related Work

Despite well-known guidelines for creating durable URIs [8], link rot remains a prevalent phenomenon of the web and hence has been subject to numerous studies. Koehler [9], for example, conducted a four-year longitudinal study where he continuously monitored the state of a random set of URIs. His results show that approximately 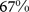 of the URIs became inaccessible after the four-year period.

The 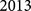 report by the Chesapeake Digital Preservation Group [10], as another example, shows that link rot is a ubiquitous problem in legal documents as well. The group reports that more than 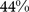 of URIs in online law- and policy-related materials published between 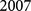 and 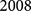 were subject to link rot in 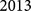. A follow-up study by Zittrain et al. [11] confirmed these findings.

The phenomenon of link rot has also been extensively studied for URI references in scholarly articles. Wren [12] extracted 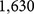 URIs from MEDLINE abstracts in 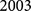 and 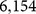 unique URIs in a follow-up study in 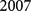 [13]. He found in both studies approximately 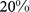 of URIs to be inaccessible. Duda et al. [14] extracted 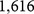 URIs from articles published in four Ecological Society of America journals and found that up to 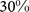 of the URIs were inaccessible. Lawrence et al. [15] observed URI references in articles in the computer science domain and found that between 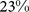 and 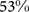 of all URIs in papers authored between 1994 and 1999 were rotten by 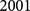. Similarly, Spinellis [16] investigated URIs referenced in articles published in “Communications of the ACM” and “IEEE Computer Society”. He found that 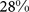 of all URIs were unavailable after five years of publication and 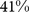 after seven years. Dimitrova and Bugeja [17] investigated the availability of URI references in five leading journalism and communication journals. They found that 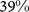 of references in articles published between 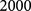 and 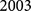 were inaccessible at the time they conducted their experiment in 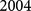. Aronsky et al. [18] obtained a random 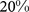 sample of articles released every day via PubMed for a 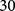 day period in 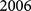. Immediately after the publication of an article they checked the online availability of all 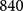 extracted URI references and found that almost 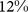 of them were already inaccessible only two days after publication. While all of these studies are related to our work, they utilize small datasets covering a narrow range of disciplines.

Dellavalle et al. [19] observed URI references in articles published in journals with a high impact factor. They discovered that about 30% of the articles they observed contained at least one URI reference. They saw the portion of URIs affected by link rot increase from 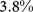 only 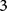 months after publication of the referencing article to 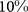 after 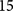 months to 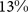 after 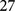 months. Hennessey and Ge [20] extracted 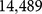 unique URIs from Thomson Reuters Web of Science paper abstracts and found 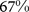 of the unique URIs still to be available. They further estimate the median lifetime of a referenced URI to be 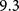 years. These two studies are particularly relevant to our work as their datasets contain articles from various disciplines. However, their scale is incomparable to that of ours.

Other related work has addressed the search for copies of missing resources either in web archives or in live web search engines. For example, Sadat-Moosavi et al. [21] investigated articles published between 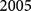 and 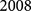 in library and information science journals in the first half of 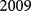. They found 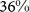 of URI references to be inaccessible but applied various intuitive URI “refinement strategies” (URI path shortening, manual editing, etc.) to search for the inaccessible URIs in the Internet Archive as well as in Google. This step decreased the inaccessibility rate of all URIs from 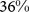 to 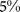. Similar approaches were taken in [22] and [23]. While these approaches demonstrate the potential value of web archives, they do not consider the temporal proximity of Mementos to the publication date of the referencing article. Further, all studies only look for Mementos in the Internet Archive whereas our work queries multiple web archives thereby improving the chances for the retrieval of Mementos.

Sanderson et al. [24] conducted a pilot study that ultimately provided the motivation for our current work. They analyzed the persistence of web resources referenced in scholarly articles from the preprint repository arXiv and the University of North Texas (UNT) scholarly repository. They found that, in the UNT corpus, 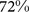 of URIs were either still available at their original URI on the live web or a Memento was available for them in a web archive, or both. This result implies, however, that 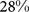 of URIs were forever lost as they were both inaccessible on the live web and archival snapshots were unavailable for them. The corresponding numbers for the arXiv corpus were 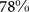 and 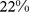. Sanderson et al. investigated the persistence of more than 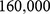 URIs which, to the best of our knowledge, was, until our current study, the largest URI corpus used to study link rot.

The dynamic nature of web resources has also been studied extensively. For example, Brewington and Cybenko [25] found that the web is fairly young. Based on their sample set, 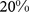 of web pages were less than 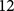 days old and 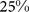 less than 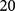 days old. They computed the mean lifetime of a page between 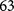 and 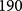 days, with the median being 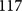 days.

Cho and Garcia-Molina [26] classified web pages simply as changed or not changed over the course of their experiment. They observed significantly different change frequencies between various top level domains (TLDs). For example, more than 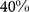 of pages in the 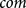 TLD changed every day but pages in 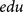 and 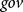 domains were more static. Interestingly, they found that it took merely 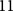 days for 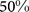 of the pages in the 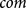 domain to change but it took almost 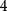 months for 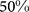 of the pages in the 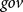 domain to change.

Fetterly et al. [27] expanded on Cho's and Molina's work and observed more than 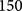 million HTML pages over a span of 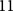 weeks. One of their main findings was that larger pages, in terms of byte size, change more often and more significantly than smaller pages. They also observed a higher link rot frequency for pages in the 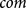 and 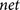 TLDs compared to pages in the 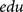 and 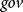 TLDs.

Ntoulas et al. [28] discovered that the frequency as well as the severity of content drift in the past is a reliable indicator for the future. This means that the rate of content drift of any given page is likely to remain consistent over time. To put it differently, if a web page changed significantly last week, it will likely change by a similarly large degree next week.

A very large fraction of changing web resources was also observed by Adar et al. [29]. In their corpus, 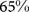 of pages displayed some degree of change and, on average, the changes occurred rather frequently, every 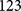 hours. They further discovered that the degree of change depends on factors such as the TLD and the popularity of the page. For example, consistent with previous work, they found that educational and government domain addresses did not change as frequently or as severely as pages in other domains. However, more popular and more frequently accessed pages changed at a faster pace.

In a related study, Adar et al. [30] investigated the structural changes of web pages. They analyzed the change frequency of Document Object Model (DOM) elements within web pages. According to their results, the median survival rate of DOM elements after one day is 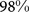. After one week it is still fairly high at 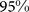 but it drops to only 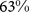 after five weeks and a mere 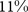 after one year.

## Methods

For our series of experiments, we generated three corpora of articles from different sources: arXiv, Elsevier, and PubMed Central (PMC). We collected articles published between the beginning of January 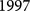 and the end of December 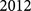. The lower temporal boundary is motivated by three considerations. First, we do not expect a vast number of articles published prior to 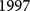 to contain URI references. Second, the world's first web archive - the Internet Archive - started its activities in early 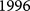 making this the earliest possible year for investigating web archive coverage for referenced URIs. Third, we know from previous work [31], [32] that the coverage in those early years of web archiving is rather sparse. Hence, the lower temporal boundary yields a realistic compromise between the availability of Mementos in web archives and the likely occurrence of URI references in published articles. The choice of December 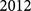 as the upper temporal boundary for the experiments that we conducted in early 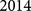 aligns with our desire to allow link rot to start manifesting itself for more recent publications.

We extracted and characterized all URI references from the articles that remained after various steps of post-processing of the three corpora. These URIs were then made subject to two tests. First, the referenced URIs were polled on the live web to determine whether the links were active or rotten. Second, multiple web archives were consulted using the Memento “Time Travel for the Web protocol” [33] to determine the archival status of the referenced URIs.

### Data Collection

We collected three corpora containing articles published within our desired time period starting January 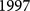 and ending December 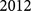. While constraining to this time period was straightforward for arXiv, the availability of multiple publication dates for Elsevier and PMC posed a challenge. Indeed, we found various dates annotated with different labels: *epreprint* for an electronic preprint, *epub* for the publication date of the electronic version, and *ppub* for the publication date of the print version. Also, we were not able to determine a consistent pattern for the temporal order of these various types of publication dates. For the sake of consistency, and aligned with the fact that our research pertains to web based scholarly communication, we decided on an approach to select an article's publication date that gives priority to dates of electronic publication: the publication date of the electronic version was given priority, followed by that of the electronic preprint, itself followed by that of the print version.

Our first corpus was generated by collecting articles from **arXiv** [34], a popular repository of preprints, covering physics, mathematics, and computer science. Through interaction with the repository administrator, we obtained all articles published in our desired time period in PDF format along with associated metadata records containing the author(s), the title, the publication date, and the arXiv-specific subject classification of each article. This amounted to a total of 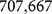 articles (Filter #4 in Table 1).

**Table 1:** Number of articles per corpus after each filtering step.

#	Filter	arXiv	Elsevier	PMC	total
1	none		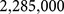	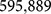	
2	no subscription		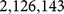		
3	API error		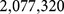		
4	desired time period ( 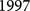 – 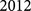 )	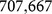	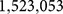	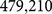	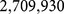
5	full-length articles	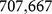	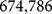	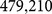	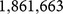
6	**STM articles**	**707,667**	**655,040**	**479,194**	**1,841,901**

Our second corpus was obtained from **Elsevier** [35], a major publisher of scholarly articles that cover a wide range of subjects. We collected Elsevier articles in a 3-step process:

1. Using the DOI randomizer Randoim [36] provided by Crossref Labs, expressing interest in only Elsevier articles published during our desired time period, we obtained almost 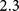 million registered DOIs.
2. Using CrossRef's Prospect API [37] (the service has since been renamed to “CrossRef Text and Data Mining”), we attempted to download the XML-formatted articles associated with the obtained DOIs. While the large majority of attempts were successful, downloads failed in cases where neither of the collaborating institutions (LANL and the University of Edinburgh) had a proper institutional subscription to Elsevier journals and in cases where the API returned an error, probably due to its beta deployment status. Slightly over 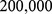 downloads failed (Filter #2 and #3 in Table 1) whereas more than 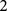 million were successful.
3. When inspecting the downloaded articles, we found that the CrossRef's Prospect API had returned articles with a publication date, selected from the articles using the aforementioned approach, that was outside our desired time range. Hence, we applied an additional filter to enforce the appropriate temporal boundary on the corpus and ended up with 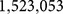 Elsevier articles (Filter #4 in Table 1).

Our third corpus was derived from **PubMed Central (PMC)** [38], an openly available full-text archive of journal articles, mostly covering biomedicine and life sciences. We downloaded the archive consisting of a total of 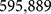 PMC articles in XML format. The publication date selected from the articles allowed us to straightforwardly narrow the archive down to our desired time period, leaving us with 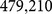 PMC articles (Filter #4 in Table 1).

### Data Post-Processing

The three corpora resulting from the Data Collection phase consisted of articles published in our desired time period but required further post-processing in order to obtain a collection that could be used for our experiments. First, in order to achieve consistency across the three corpora, only full-length articles were considered. Second, in order to allow extrapolation of our findings on the basis of available annual publication rates of Science, Technology, and Medicine (STM) articles, only articles that pertain to this denomination were selected.

The former filter only had to be applied to the Elsevier corpus as it was the only one to contain several publication types labeled as, for example, full-length article, abstract, short communication, discussion, correspondence, and book review. Publication types other than full-length article were filtered out, leaving us with 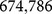 articles (Filter #5 in Table 1).

The latter filter was not applied to arXiv as its subjects fall inarguably into the STM domain. However, the restriction to STM articles was applied to both the Elsevier and PMC corpora. For both, the article XML contains the ISSN of the journal the article was published in. We obtained ISSN to subject mappings from the Ulrich's Knowledgebase [39] and retained only articles published in journals that had at least one subject classification that aligns with the STM designation used in the report on Science and Engineering Indicators 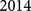 [40]. After applying this filter, 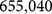 articles remained for the Elsevier corpus and 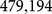 for PMC (Filter #6 in Table 1).

### URI Extraction and Characterization

We then set out to extract all URI references from these corpora, not just the ones in each article's reference section but also those in the abstract, the body, and the footnotes. Two of the corpora consist of articles in XML format but the articles in the third corpus, arXiv, are in PDF. In order to have these arXiv articles in an accessible format, they were converted to XML using the popular command line tool *pdftohtml* [41]. This conversion led to challenges for the URI extraction process such as the recognition of URIs that were separated by a newline in the PDF, and handling of URIs for which an underscore is represented as an image in the PDF instead of as a character. A detailed description of the extraction algorithm, its handling of these challenges, and its precision/recall performance as compared to other common URI extraction mechanisms is provided in [42].

Since our experiments are about investigating the current and archival status of referenced URIs, we only maintained articles that contain URI references (Row #2 in Table 2). We then proceeded to map these URI references into three categories:

**Table 2:** STM articles per corpus and the categories of URI references they contain.

#	STM articles	arXiv	Elsevier	PMC	total
1	total	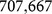	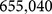	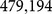	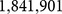
2	with URI references	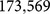	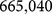	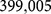	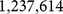
3	with excluded URI references	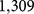	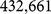	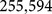	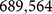
4	with URI references to articles	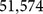	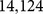	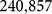	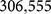
5	**with URI references to web at large**	**142,134**	**94,645**	**156,160**	**392,939**

- References to web at large resources, which are the focus of our research.
- References to journal articles, which are outside the scope of our research.
- References that should be excluded because they would skew the results of live web and web archive lookups, for example, syntactically invalid URI references.

Intuitively, one would assume that URI references to journal articles can readily be recognized by detecting HTTP URIs that carry a DOI, e.g., http://dx.doi.org/10.1007/s00799-014-0108-0. However, it turns out that references rather frequently have a direct link to an article in a publisher's portal, e.g. http://link.springer.com/article/10.1007%2Fs00799-014-0108-0, instead of the DOI link. As a result, URI references to scholarly articles were recognized as follows:

- URIs starting with http://dx.doi.org.
- URIs that result in a match against CrossRef's reverse domain lookup [43], which allows testing whether the base URI of a URI reference is associated with a CrossRef member.
- URIs with a hostname that is associated with a scholarly publisher on the basis of a manually compiled list of publisher base URIs, including, for example, arXiv.org.

The following URI references were excluded for the purpose of our research:

- URIs that are not part of the actual article but are added by the publisher. The Elsevier corpus contains numerous such links to support navigation in its portal and a majority of the articles in the PMC corpus contain a link to a license. The vast majority of excluded URI references falls into this category.
- URIs with a URI scheme other than *HTTP* or *HTTPS*.
- URIs that do not have a top level domain (TLD) registered with the Internet Assigned Numbers Authority (IANA) [44] (We used version 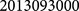, last updated on 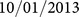 of the list of TLDs).
- “dummy URIs” such as *example.com*, *foo.bar*, and *localhost*.
- URIs with domain names expressed as IP addresses that are known to not have a global reach, such as *127.0.0.1*, and the ranges *192.168.x.x*, *172.16.x.x* - *172.31.x.x*, and *10.x.x.x*, according to RFC 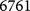 [45].

URI references that were not excluded in this manner and that were not references to journal articles were then considered to be references to web at large resources. The result of this URI categorization per corpus is shown in Table 3, whereas Table 2 shows the number of articles that contain the respective categories of URI references. As can be seen, close to 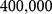 articles across the three corpora (Row #5 in Table 2) contained over a million web at large references (Row #4 in Table 3) - the combination of a referenced URI and the selected publication date of the referencing article - that became the subject of our investigations.

**Table 3:** Characterization of URI references in STM articles per corpus.

#	URI references	arXiv	Elsevier	PMC	total
1	total	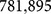	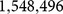	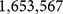	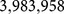
2	excluded	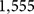	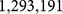	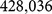	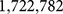
3	to articles	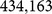	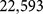	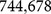	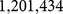
4	**to web at large**	**346,177**	**232,712**	**480,853**	**1,059,742**

### Live Web Lookup of URI References

In order to be able to quantify the extent of link rot in scholarly articles, we tested the status of each URI reference on the live web. This was done in the course of March 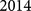.

We used the command line tool *cURL* to send an HTTP GET request against each URI and recorded the HTTP response code. Our initial attempts used the more performant HTTP HEAD request but we resorted to HTTP GET after discovering that web servers frequently respond differently to HEAD and GET requests, and, in certain cases, do not support HTTP HEAD at all. In order to avoid being trapped in loops, we configured cURL to follow a generous maximum of 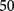 HTTP redirects (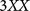-level HTTP response codes).

Per URI, this test had two possible outcomes. On the one hand, the HTTP transaction chain could end successfully with a 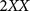-level HTTP response code. In this case we declared the URI to be **active** on the live web. On the other hand, the transaction chain could end unsuccessfully with a response code other than 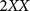-level or with a chain for which the maximum number of redirects had been reached. In this case we declared the URI as being inactive and, hence, the link as being **rotten**, subject to link rot. In order to account for possible transient errors, we revisited all URIs that fell into the rotten category two weeks after the initial attempt and changed their status from rotten to active if the response was successful in the second run.

### Web Archive Lookup of URI References

In order to be able to quantify the archival coverage for referenced URIs, we used the Memento protocol [33] and associated infrastructure to programmatically check for Mementos of each URI reference in web archives. The Memento protocol extends the HTTP protocol with datetime negotiation, a variant of content negotiation. A Memento client requests an old version of a resource by expressing the resource's original URI along with the datetime of the version it is interested in. A Memento server, such as a web archive, responds with an archived snapshot of the resource that is temporally closest to the datetime requested by the client. A Memento Aggregator simultaneously polls multiple web archives and returns the temporally closest snapshot available irrespective of the archive it resides in.

For our web archive lookup, we used six publicly accessible web archives: Internet Archive, archive.is (since renamed to archive.today), Archive-It, British Library Web Archive, UK National Archives Web Archive, and the Icelandic National Archive. There are two motivations for the choice of these archives. First, they are all natively compliant with the Memento protocol making the lookup of a million URIs both feasible and scalable. Second, consistent with the findings in [46], we observed during test runs that the vast majority of Mementos was provided by the pool of these six web archives and therefore felt that the marginal return on investment that would result from polling additional archives did not justify the additional resources required.

Since we are interested in revisiting referenced resources in the state they were when the referencing article was published, we used the Memento protocol to request a snapshot of each referenced URI, expressing the selected publication date of the referencing article for datetime negotiation. This protocol request was issued against a special-purpose Memento Aggregator that covered the aforementioned six archives. If a request failed to return a Memento, we declared the URI reference **not archived**. If a request returned a Memento, according to the Memento protocol, it had to be the one with an archival datetime that was temporally closest to the selected publication date of the referencing article. Since web archives also archive HTTP responses other than successful 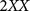-level ones, we then performed a test similar to the one for the live web but this time against the web archive that had provided the Memento. The Memento's URI was dereferenced and a maximum of 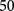 HTTP redirects (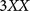-level HTTP response codes) were followed. If the HTTP transaction chain resulted successfully with a 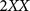-level HTTP response code, we designated the URI reference as **archived** and stored the Memento's URI, its archival datetime, and the time difference between that archival datetime and the selected publication date of the referencing article. If the HTTP transaction chain was longer than the set maximum or if the chain ended with a HTTP response code other than a 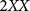-level, we declared the URI reference as **not archived**.

## Results

### Articles and URI References

We proceeded by trying to obtain a better insight into the results of our categorization of extracted URI references and the articles they were extracted from. We did not consider the excluded URI references in this analysis because the overwhelming majority of those are references that are not an integral part of the articles but rather added post-publication by the publisher. Hence, we only considered URI references to articles and to web at large resources.

**Figure 1:**
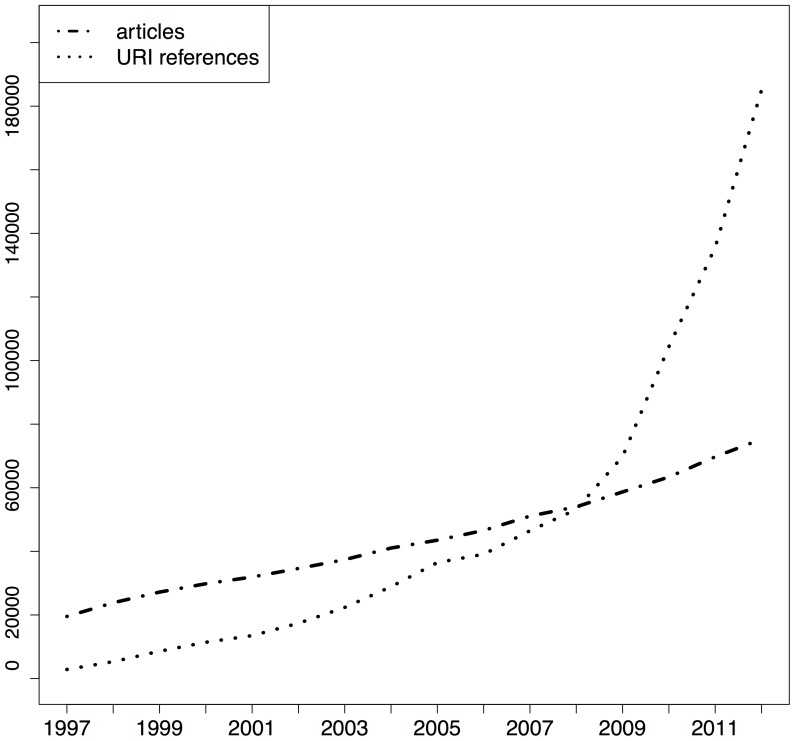
STM articles and URI references per publication year - arXiv corpus.

**Figure 2:**
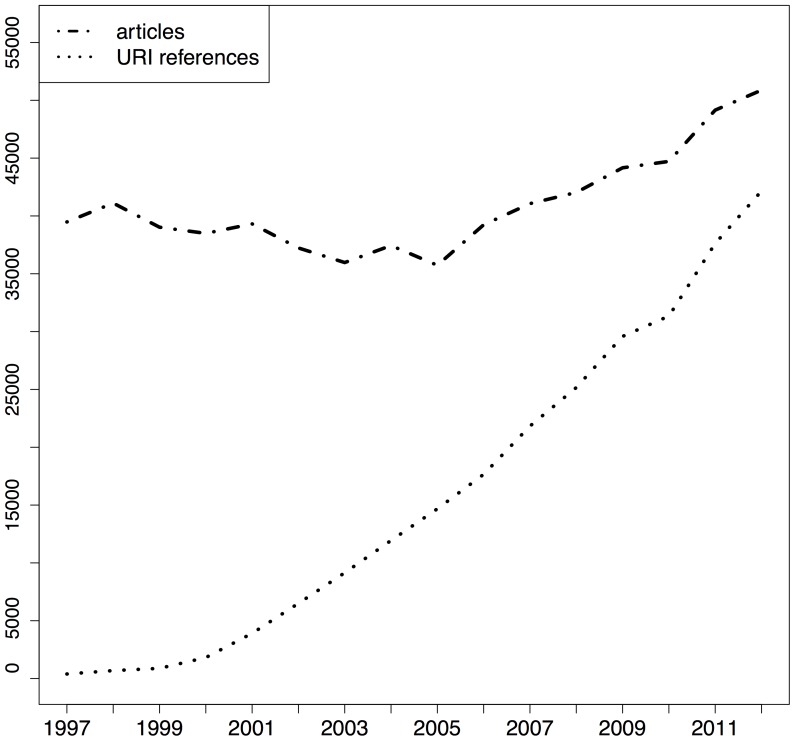
STM articles and URI references per publication year - Elsevier corpus.

**Figure 3:**
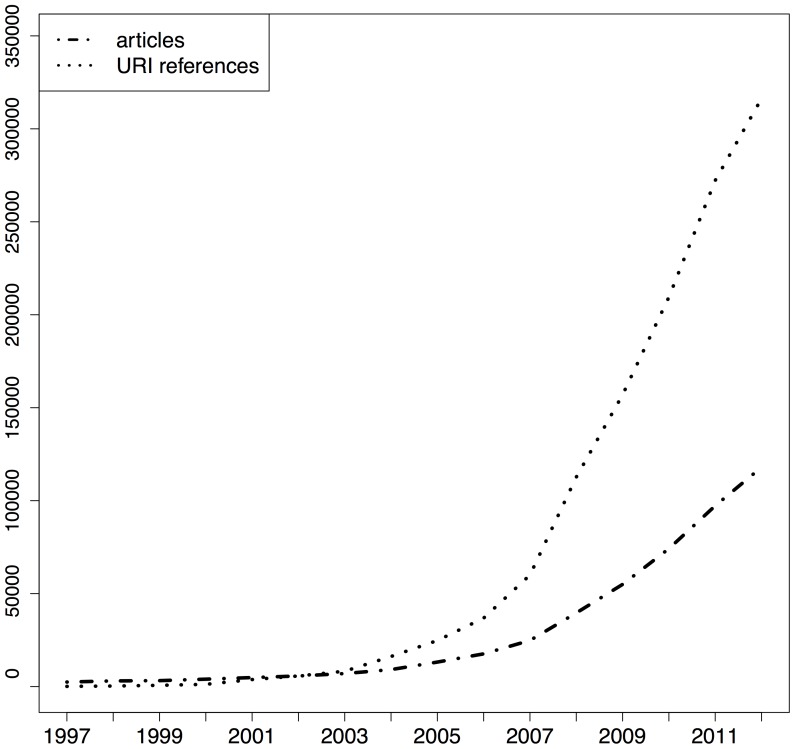
STM articles and URI references per publication year - PMC corpus.

Figs. 1, 2, and 3 show the total number of articles per publication year for the respective corpora (dot dashed lines) as well as the corresponding number of URI references in those articles (dotted lines), the sum of URI references to articles and to web at large resources. All corpora show a growth in the number of articles published per year. The PMC corpus is very small for early publication years but it picks up, initially slowly around 2005, and then rather abruptly around 2007. This growth pattern is related to the PMC submission policy that changed from voluntary to mandatory in 2008 [47] and which resulted in a dramatic growth in submissions from there onwards [48]. The growth of arXiv articles is not as sudden and explosive as that of PMC, yet clearly faster than that of Elsevier. This growth pattern is likely related to the extension of arXiv to disciplines other than the original physics subject matters as well as the growing popularity of communication by means of preprints, which itself is likely related to an increasing interest, during the past decade, in open access to scholarly literature. The same figures also show that, across the three corpora, the number of URI references contained in articles grows from hardly any in 1997 to a substantial number in recent years. Also, the pace of growth for URI references is substantially higher than for articles. Understanding from Table 2 that many articles do not contain URI references of the considered types at all, this means that the ones that do contain them use them generously. The growth pattern for URI references in arXiv stands out in that, initially, they grow at a pace similar to articles but, around 2008, pick up rapidly and start showing a growth pattern similar to that of URI references in the other corpora.

**Figure 4:**
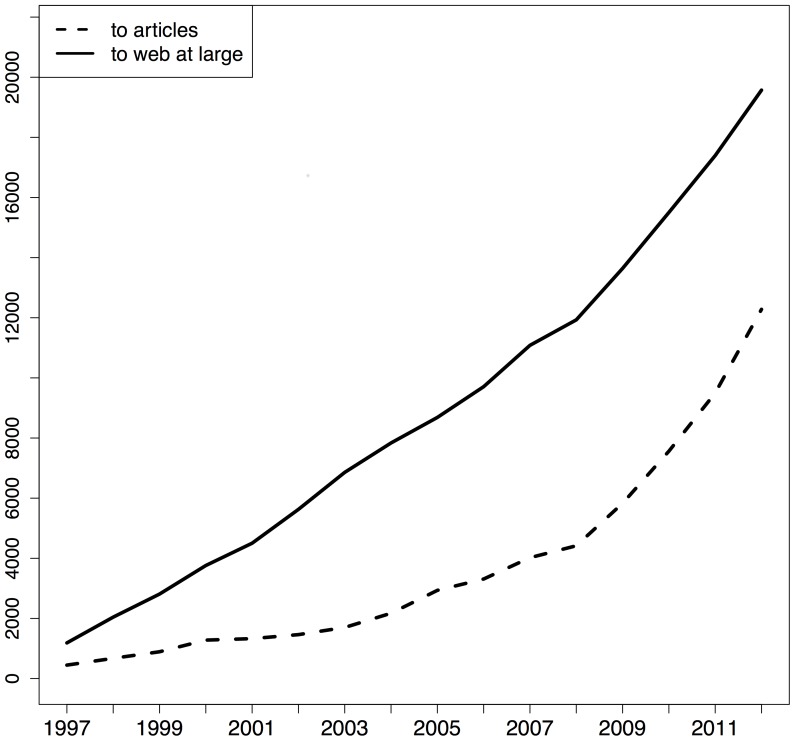
STM articles per URI reference type they contain and per publication year - arXiv corpus.

**Figure 5:**
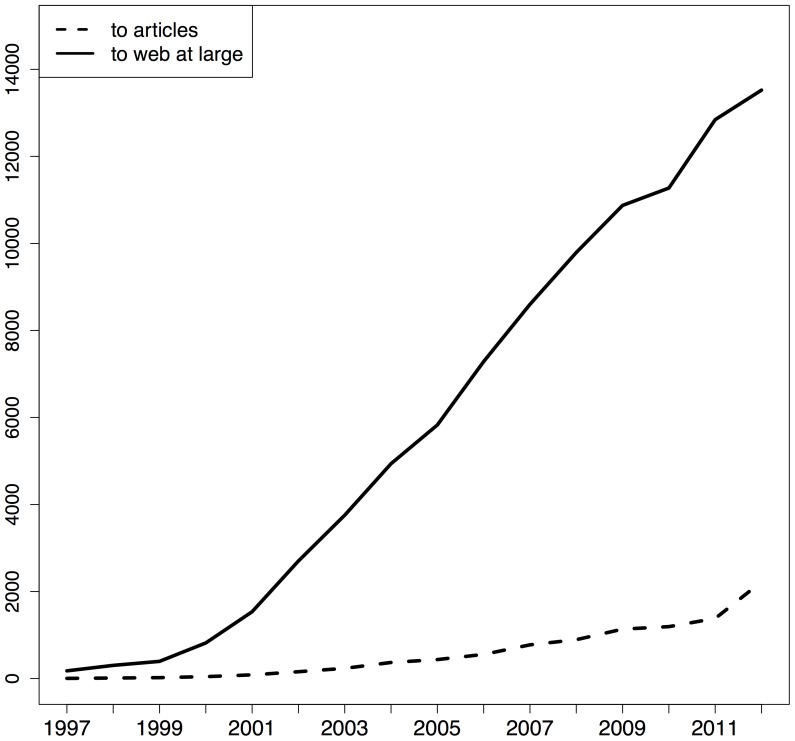
STM articles per URI reference type they contain and per publication year - Elsevier corpus.

**Figure 6:**
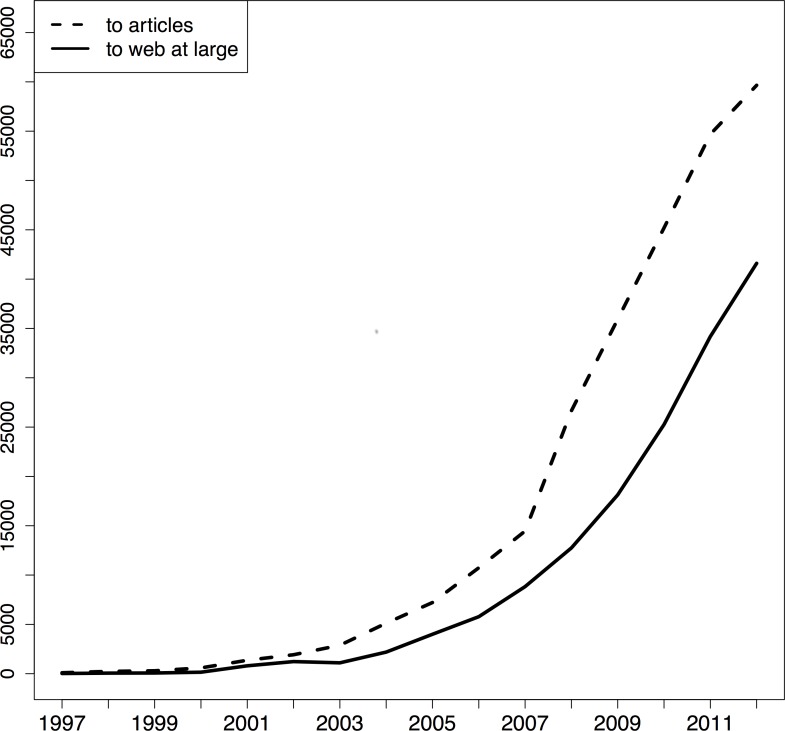
STM articles per URI reference type they contain and per publication year - PMC corpus.

Figs. 4, 5, and 6 provide, per corpus, the number of articles per publication year that contain URI references to articles and to web at large resources, respectively. In these figures, the solid line pertains to articles with web at large references and the dashed line to articles with URI references to other articles. For arXiv and PMC, the growth rate for articles that contain these two types of references is similar but, over the entire time span arXiv contains more references to web at large resources whereas PMC contains more URI references to articles. Fig. 5 shows hardly any URI references to articles. This is due the fact that, in the Elsevier XML that we parsed, article references are expressed in purely textual form; they do not contain the HTTP URI variant of DOIs, neither the DOI itself. Elsevier seems to add these identifiers on the fly when rendering articles in its portal.

**Figure 7:**
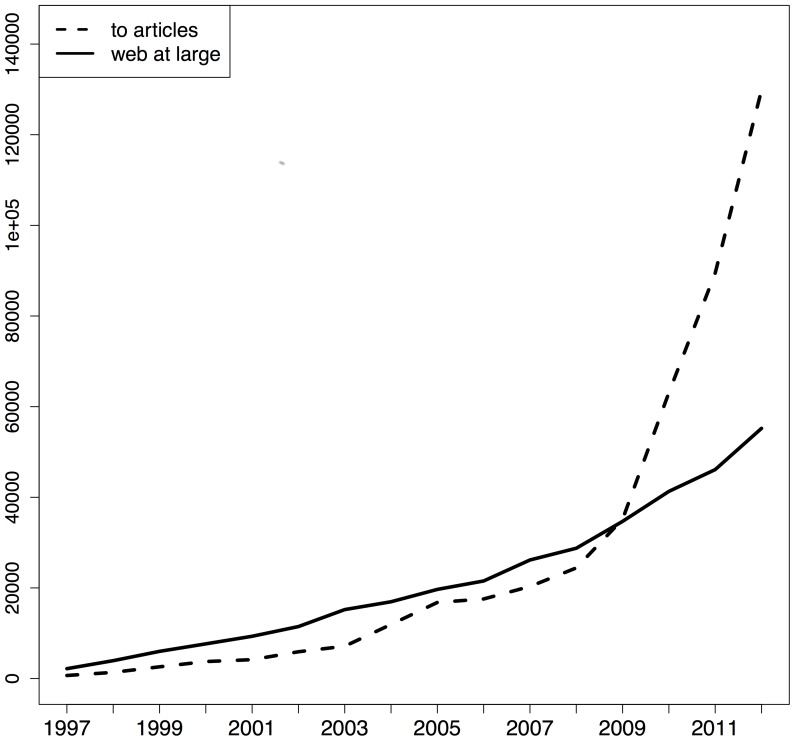
URI reference type per publication year of the referencing STM article - arXiv corpus.

**Figure 8:**
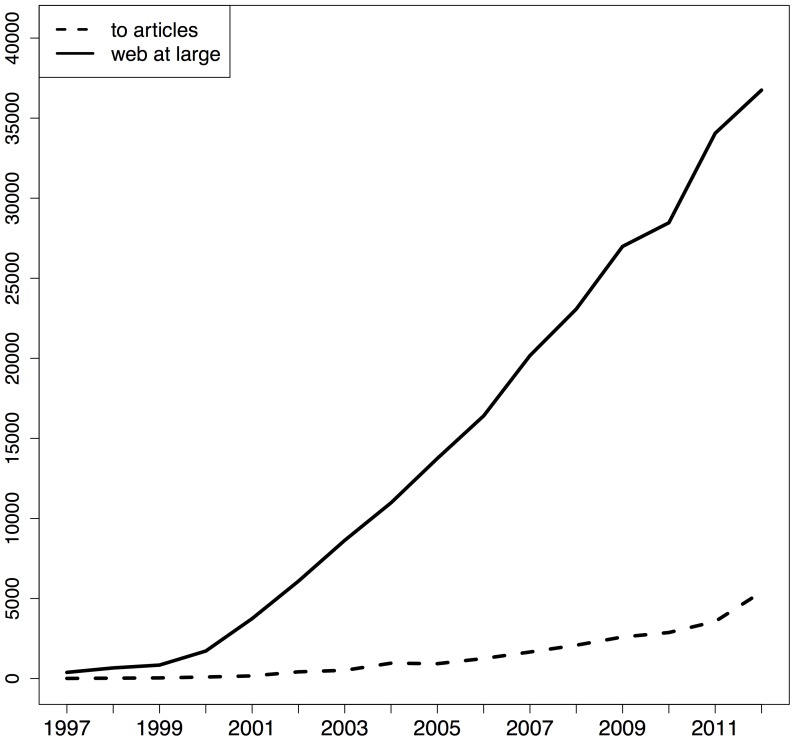
URI reference type per publication year of the referencing STM article - Elsevier corpus.

**Figure 9:**
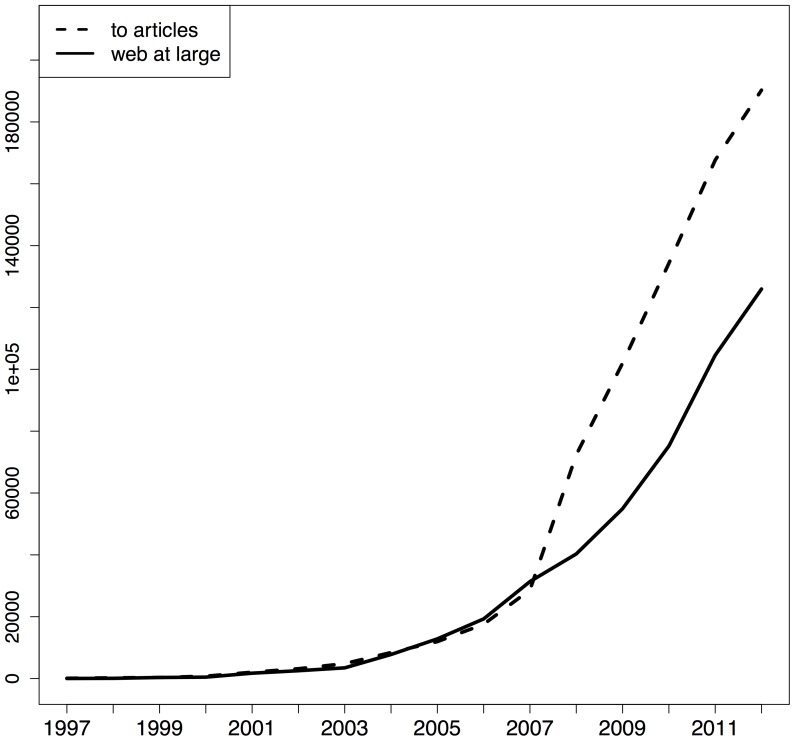
URI reference type per publication year of the referencing STM article - PMC corpus.

Figs. 7, 8, and 9 provide, again per corpus, an overview of the number of URI references per type and per publication year of the referencing article. In these figures, the solid line pertains to web at large references and the dashed line to URI references to articles. The dashed line in Fig. 8 is an artifact of the textual nature of article references in Elsevier's XML. Across the three corpora, a clear growth in the number of references to web at large resources can be observed. The figures for arXiv and PMC indicate that both types of reference initially grow at the same pace but that, around 2008, URI references to articles start to grow faster than references to web at large resources. This is probably related to the overall trend to use the HTTP variant of DOIs in references that seems to have started around that time, and which eventually led CrossRef to recommend that notation [49]. Despite this dominance of URI references to articles, these figures show that references to web at large resources continue to grow at a steady pace and add up to a substantial amount for recent publication years.

### Link Rot - Revisiting Current Context

**Figure 10:**
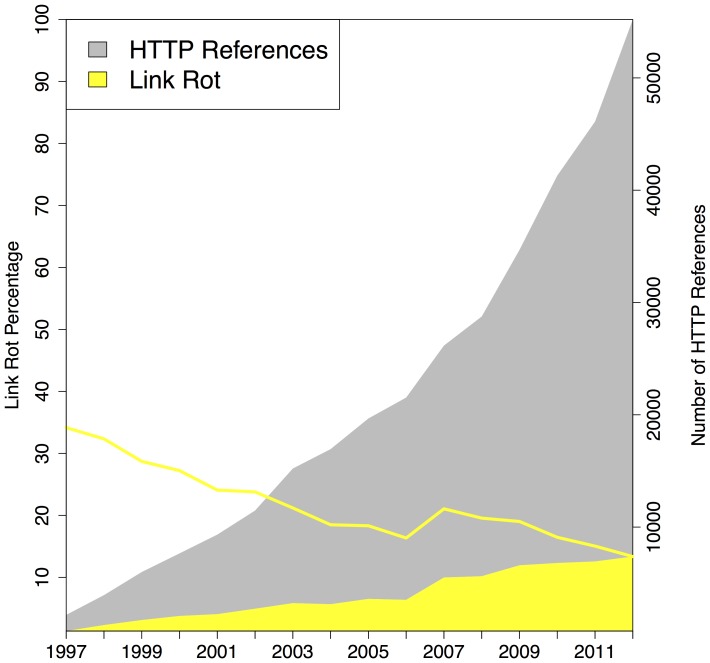
Link Rot - arXiv corpus.

**Figure 11:**
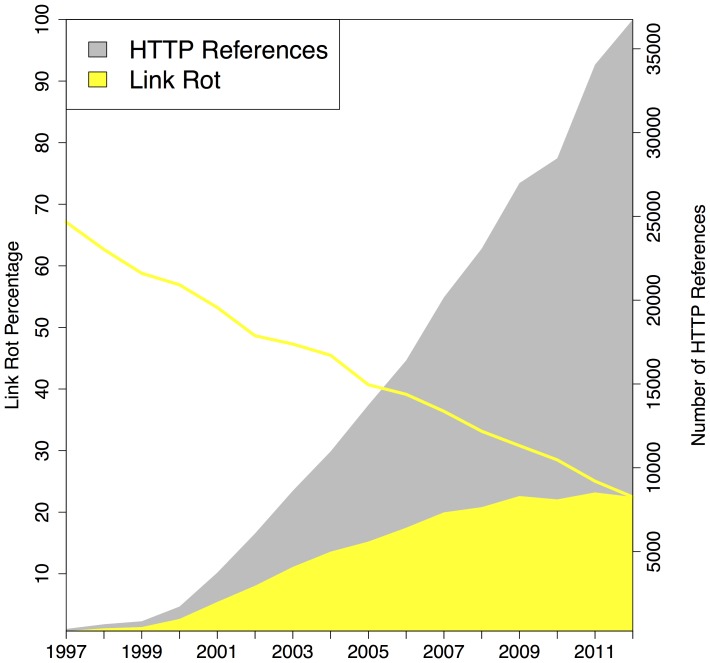
Link Rot - Elsevier corpus.

**Figure 12:**
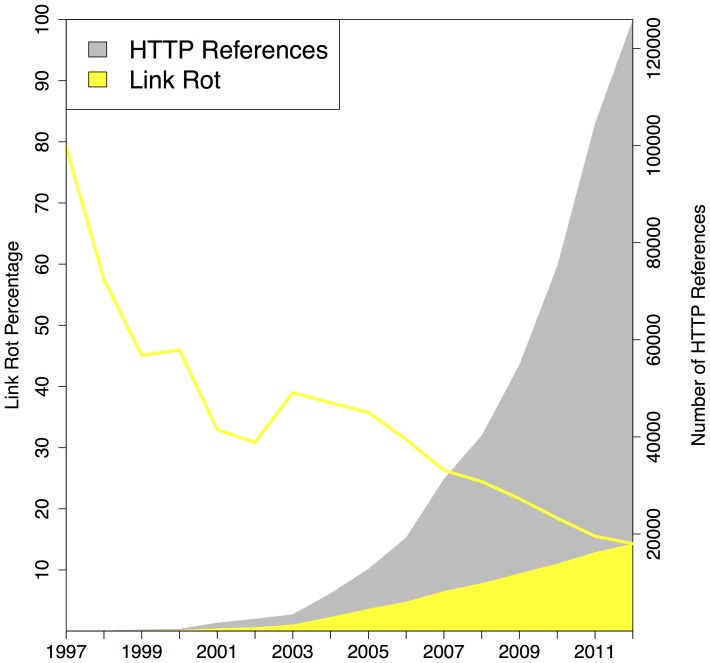
Link Rot - PMC corpus.

Our next experiment was aimed at quantifying link rot for all three corpora. For this purpose we considered the number of URI references to web at large resources in function of the selected publication date of the referencing article, and did the same for the URI references that were classified as rotten when being polled on the live web. Figs. 10, 11, 12 displays the extent of link rot for the three corpora: the gray surface of the plots shows the total number of references to web at large resources, the yellow surface shows the total number of references affected by link rot. The right hand side y-axis shows the actual numbers. The solid yellow line in the same figures, to be interpreted using the left y-axis, shows the percentage of rotten URI references in function of the selected publication date of the referencing article.

All three corpora show a moderate, yet alarming, link rot ratio for references made in recent articles, published in 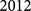: 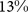 for arXiv, 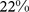 for Elsevier, and 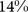 for PMC. Not surprisingly when considering the dynamic nature of the web, for older articles the link rot ratio increases in all corpora. For publication year 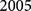, the link rot ratio stands at 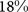, 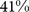, 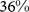 for arXiv, Elsevier, and PMC, respectively. Going back to the earliest publication year in our corpora, 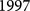, the ratios become 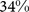, 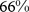, and 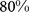, respectively. The latter ratio should be taken with a grain of salt considering that, up until 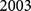, the PMC corpus annually only has a few hundred URI references. The three corpora exhibit a trend found in all link rot studies to date: link rot manifests itself increasingly as links age. However, the extent to which the corpora suffer from link rot is distinct. Inarguably, over time, arXiv exhibits the lowest link rot ratio, and maintains an impressively low rate even for the oldest publication years. And, when discounting the publication years prior to 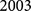 because PMC hardly contains any URI references then, the link rot ratio for Elsevier is significantly worse than that for PMC. Section **Loss of Current and Past Context**, provides some insights into why these corpora exhibit different link rot patterns.

### Archival Status - Revisiting Past Context

The only recourse for determining what content was provided at the end of a URI reference that is rotten is to look that URI up in web archives to try to find Mementos for it. But, web archives are also essential when trying to revisit the content that was originally referenced by URI references that remain active to date. Indeed, we know from the research literature that, generally, web resources are subject to content drift [9], [25]–[30]. Hence, it is fair to deduce that web resources that are referenced in journal articles are also subject to content drift. This deduction is supported by two strong indications that, when it comes to our problem domain, URI references made in journal articles are very similar to references made on the web in general. First, results from various link rot studies that target the web in general, on the one hand, or the scholarly literature, on the other, reveal that URI references in both cases exhibit a very similar link rot pattern [9], [15], [17], [21]. Second, content drift has explicitly been described in the literature for several of the TLDs that are targeted by URI references to web at large resources [26], [27], [29] as listed in Section **Loss of Current and Past Context**.

Therefore, in this experiment, we set out to establish an insight into the extent to which coverage of URI references in web archives may be representative of the content that was originally referenced. To that end, we use the temporal delta information gathered during the Web Archive Lookup phase. This delta is the time difference between the selected publication date of an article that references a URI and the archival datetime of the Memento for that URI that is temporally closest to that publication date. Understanding that all web resources change over time, our plausible intuition is that Mementos become more representative of the originally referenced content as the delta gets smaller. From the literature, we know that the pace at which a resource changes depends on the resource type [50] and also on the TLD, for example, 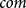, 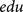, 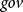 TLDs evolve at a different pace [26], [27], [29]. In an ongoing research effort we aim at providing an insight in the pace of change that is most characteristic for URI references made in scholarly publications. To that end, we are collecting a Memento of each referenced URI as soon as an article gets published and revisit those URIs on a monthly basis to determine the extent to which they evolve. Lacking such information at the time of the research reported here, we considered three deltas − 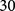 days, 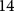 days, and 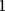 day - surrounding the article's publication date and assume that a Memento with a 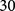 day delta is less representative of the originally referenced content than a Memento with a 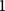 day delta.

**Figure 13:**
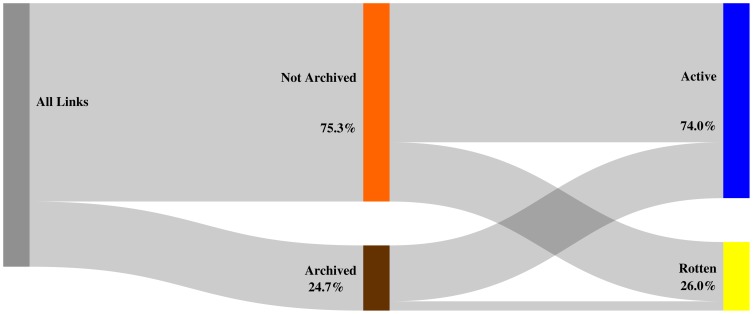
Mementos for URIs archived within 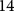 days of being referenced - arXiv corpus.

**Figure 14:**
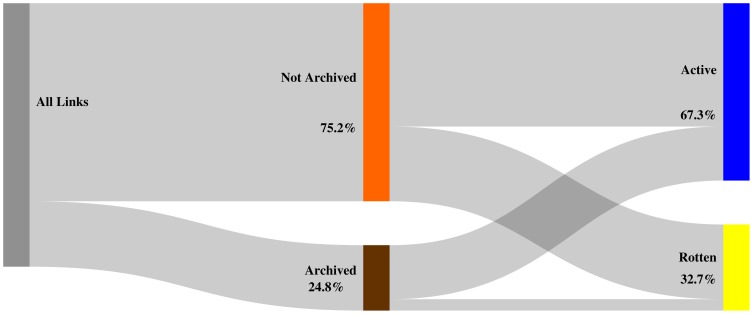
Mementos for URIs archived within 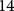 days of being referenced - Elsevier corpus.

**Figure 15:**
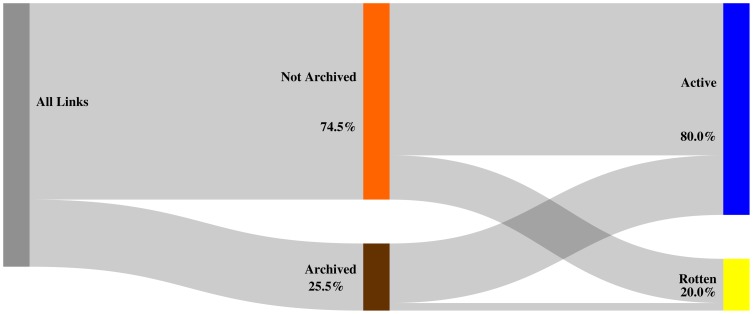
Mementos for URIs archived within 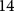 days of being referenced - PMC corpus.

Figs. 13, 14, and 15 depict, per corpus, our findings for the 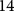 day delta. Each figure labels referenced URIs as **archived** (brown bar) if a Memento exists for that delta and as **not archived** (orange bar) when no Memento exists for it, including when no Memento exists at all. Each figure also represents the extent to which references designated as archived or not archived are **active** (blue bar) or **rotten** (yellow bar) on the live web. The hight of the colored bars corresponds with the ratio of referenced URIs that fall into the bar's category.

We find that 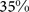 of all URI references in arXiv articles have Mementos archived within the 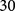 day delta. Logically, the ratio gets worse as the delta decreases: less than 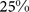 for the 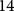 day delta (Fig. 13) and a meager 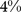 for the 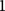 day delta. The Elsevier and PMC corpora exhibit a similar pattern with both having representative Mementos for the 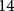 day delta for about a quarter of the URI references (Figs. 14 and 15). Generally, the lack of representative Mementos for all considered deltas is consistent across the corpora. Around 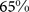 of URI references lack a Memento within the 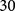 day delta and around 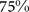 within the 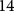 delta. Hardly any Mementos exist for the 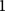 day delta, which is the delta that provides strong guarantees that a Memento would very closely resemble the originally referenced content. Figures covering the 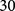 day and 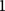 day delta are provided in the Supporting Information section.

### Loss of Current and Past Context

In this experiment, we aim at providing an insight into the loss of current and past context surrounding articles in our corpora as a result of, respectively, link rot and lack of temporally representative Mementos for referenced URIs. We considered the network consisting of individual articles and the specific URIs they reference and found that it was made up of numerous unconnected clusters and hence would not be very helpful for our purpose. Instead, we explored a more abstract network consisting of the corpora as sources of URI references and the TLD for each of those references as targets. The number of URI references to web at large resources sourced per corpus is known (Table 3) and the number of URI references targeting a TLD was obtained by simply reducing each referenced URI to its TLD. In order to keep the visualization of the resulting network interpretable, we then restricted our analysis to the top six target TLDs for our corpora: 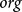, 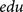, 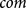, 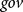, 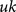, and 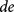.

**Figure 16:**
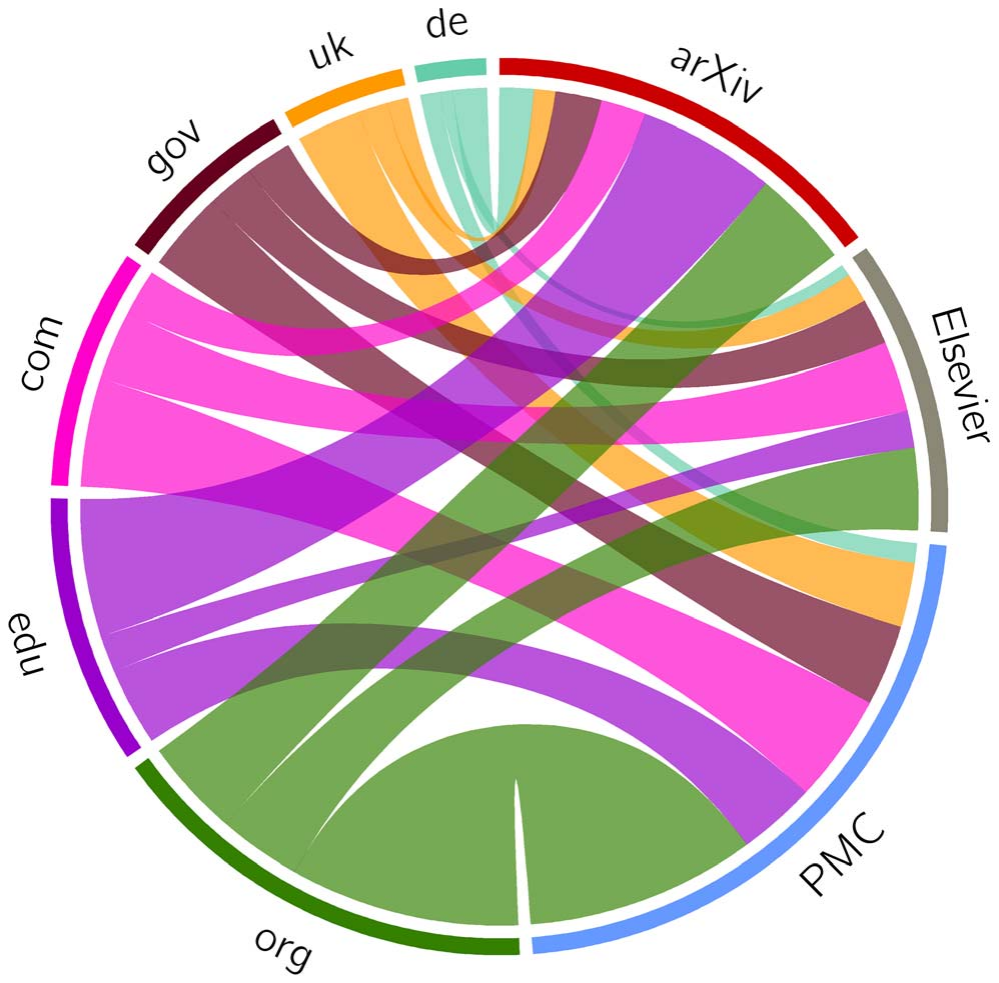
URI references: corpora as sources, TLDs as targets - all links.

Fig. 16 shows the references flowing from our three corpora on the right to the six TLDs on the left. The width of the connections is proportional to the number of references. The figure reveals that all three corpora have a large fraction of their URI references targeting resources in the 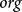 TLD. In addition, the majority of arXiv references lead into the 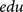 domain. Given the scholarly nature of the sources of the references, neither observation is surprising. We also observe that a significant portion of the Elsevier and PMC references point at resources in the 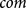 TLD while very few arXiv resources do. Since prior research [26], [29], [51] found that 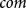 resources are significantly more prone to link rot than resources in the 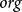 and 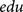 TLDs, this may explain why link rot for arXiv is, overall, less severe than for Elsevier and PMC.

**Figure 17:**
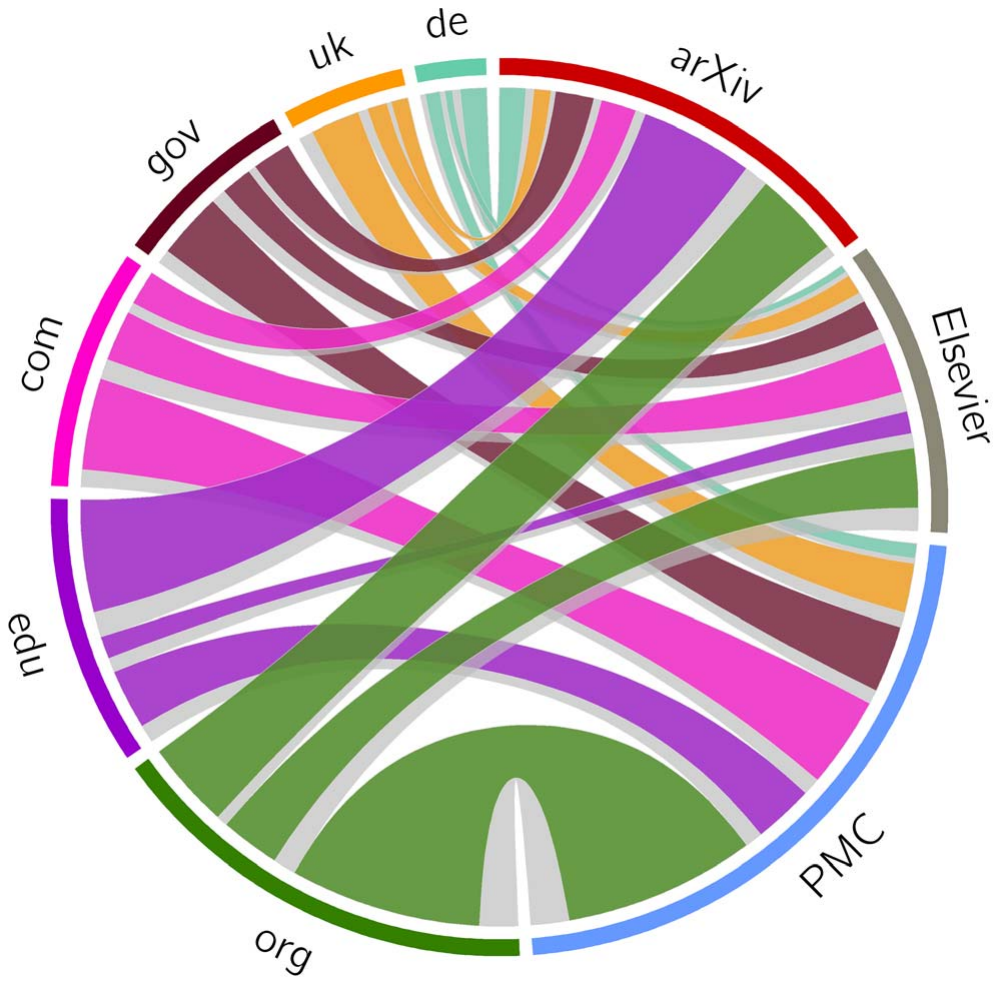
URI references: corpora as sources, TLDs as targets - active links.

**Figure 18:**
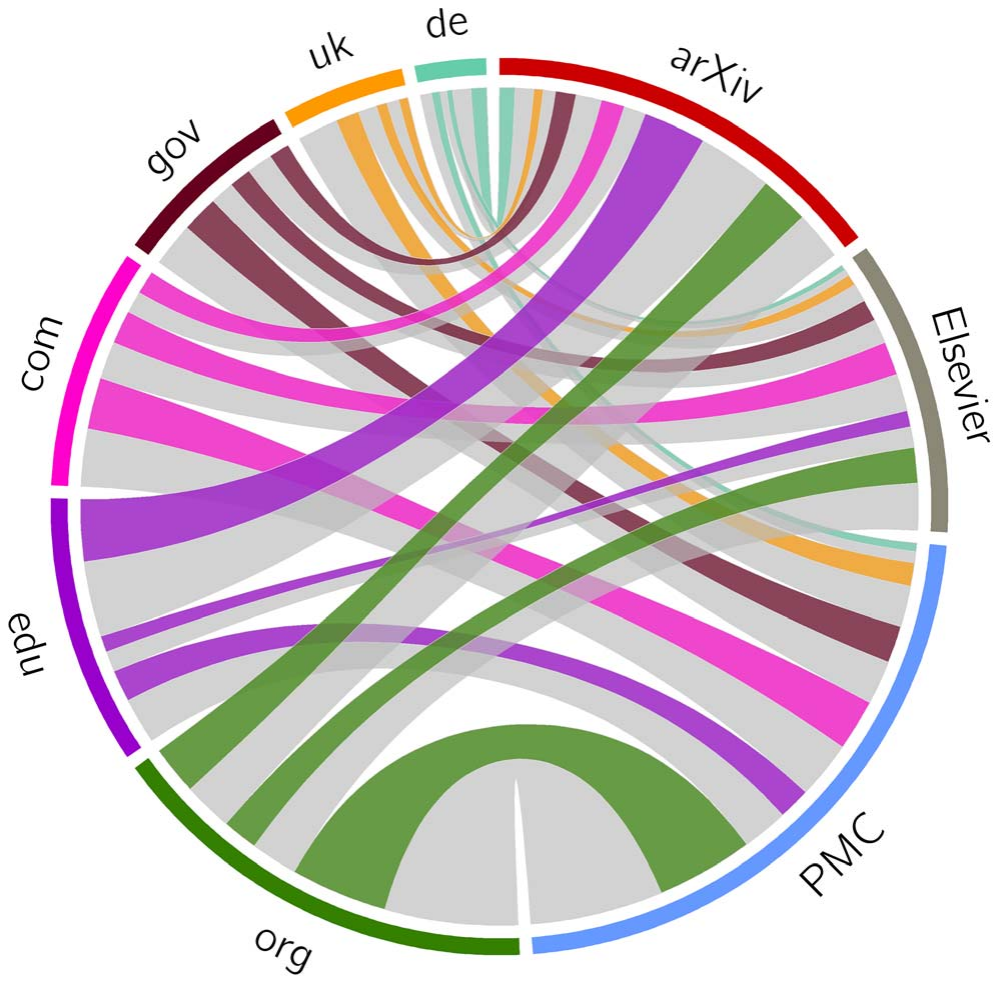
URI references: corpora as sources, TLDs as targets - Mementos created within 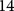 days of referencing.

Fig. 17 revisits Fig. 16 but shows the active references in color and the rotten ones in gray, thereby illustrating the extent of the loss of current context in the network. To illustrate the extent to which the past context, consisting of originally referenced content, is likely lost, Fig. 18 shows those URI references for which Mementos exist in color, and those for which Mementos are lacking (according to the 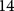 day delta of representativeness) in gray. The figure reveals that revisiting the originally referenced content that surrounded an article by means of representative Mementos in web archives is to a rather limited extent possible using that delta. Understandably, a similar figure for the generous 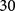 day window paints a more positive picture, whereas a figure for the stringent 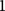 day delta reveals that revisiting the past context is largely impossible. Both figures are provided in the Supporting Information section.

### Quantifying Reference Rot for the Corpora

So far, our investigations have been based on our collection of URI references. Understanding that these references are subject to reference rot, we have assessed whether they are still active on the live web and whether temporally representative Mementos exist in web archives. In doing so, we have provided an insight into the extent to which the current and past context surrounding our corpora can be revisited. We now turn to our three corpora and consider the status of their articles with regard to reference rot by introducing the following typology:

• An article is **immune** to reference rot if it contains no references to web at large resources.
• An article is **healthy** when all its references to web at large resources are active and when a representative Memento exists for each of the referenced resources.
• An article is **infected** by reference rot if one or more of its URI references is either rotten or has no associated representative Memento.

For the purpose of this analysis, we selected the 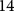 day delta surrounding the article's selected publication date as the period within which a Memento is considered representative.

Table 4 shows the total number of articles per publication year for arXiv as well as the number of articles with URI references to web at large resources. For those articles with URI references, it shows the number that contain one or more URIs that are subject to link rot and the number that contain one or more URIs that have no representative Memento for our chosen delta. Finally, it shows the number of articles infected by reference rot according to the aforementioned criterion. The table shows, for example, that 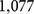 out of the 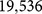 articles published in 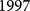 - about 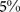 - are infected by reference rot. For publication year 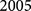 more than 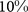 of articles are infected and for 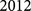 this ratio has doubled again to become 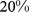. The corresponding numbers for the Elsevier corpus are shown in Table 5. As can be seen, the reference rot ratio increases from being negligible in 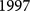 to more than 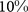 in 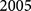 and around 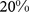 in 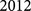. The numbers for PMC, shown in Table 6, also reveal very little reference rot for early publication years but, for more recent years, a ratio that is higher than that of arXiv and Elsevier. For example, 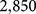 out of 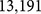 articles published in 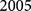 are infected, and for 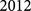 the number grows to 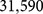 out of 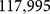, which amounts to a ratio of almost 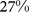. A noteworthy observation across the three corpora is the very significant overlap between articles with link rot and those for which representative Mementos are lacking. Very few articles suffer from the former but not from the latter. As a result, the number of articles infected with reference rot is very close to the number of articles that lack appropriate Mementos for referenced resources. A visualization of this data for all three corpora is provided in the Supporting Information section.

**Table 4:** arXiv articles with link rot, lacking representative Mementos, infected by reference rot.

	1997	1998	1999	2000	2001	2002	2003	2004
arXiv articles	19,536	23,841	27,188	29,815	31,936	34,613	37,405	41,040
with URIs	1,186	2,043	2,810	3,760	4,502	5,627	6,858	7,836
subject to								
Link rot	514	891	1,164	1,494	1,642	1,950	2,322	2,281
Not archived	1,076	1,856	2,530	3,133	3,446	4,270	4,718	4,946
Reference rot	1,077	1,860	2,534	3,138	3,464	4,293	4,740	4,981

	2005	2006	2007	2008	2009	2010	2011	2012
arXiv articles	43,515	46,572	51,094	54,020	58,691	63,383	69,734	75,284
with URIs	8,685	9,708	11,083	11,930	13,641	15,501	17,394	19,570
subject to								
Link rot	2,382	2,586	3,601	3,819	4,107	4,384	4,545	4,711
Not archived	5,826	6,390	7,217	8,848	11,184	12,804	13,229	15,516
Reference rot	5,867	6,425	7,251	8,869	11,198	12,813	13,246	15,531

**Table 5:** Elsevier articles with link rot, lacking representative Mementos, infected by reference rot.

	1997	1998	1999	2000	2001	2002	2003	2004
Elsevier articles	39,480	41,110	39,027	38,494	39,314	37,232	35,971	37,439
with URIs	177	303	395	819	1,536	2,702	3,755	4,938
subject to								
Link rot	141	207	271	517	912	1,582	2,188	2,791
Not archived	169	277	363	624	1,163	2,105	2,581	3,280
Reference rot	169	278	364	626	1,170	2,117	2,603	3,326

**Table 6:** PMC articles with link rot, lacking representative Mementos, infected by reference rot.

	1997	1998	1999	2000	2001	2002	2003	2004
PMC articles	2,459	3,065	3,212	4,000	4,864	5,723	7,022	9,150
with URIs	20	59	71	152	800	1,229	1,101	2,190
subject to								
Link rot	16	39	41	98	194	373	658	1,291
Not archived	18	56	55	130	643	855	765	1,344
Reference rot	18	57	55	130	644	860	769	1,364

	2005	2006	2007	2008	2009	2010	2011	2012
PMC articles	13,191	17,618	24,836	39,638	54,976	74,188	97,257	117,995
with URIs	3,998	5,780	8,829	12,753	18,132	25,260	34,184	41,602
subject to								
Link rot	2,263	3,045	4,370	5,464	6,937	8,470	10,190	11,342
Not archived	2,821	3,973	5,910	9,679	14,352	20,577	25,439	31,544
Reference rot	2,850	4,009	5,934	9,699	14,382	20,600	25,491	31,590

### Extrapolating Reference Rot to STM Articles

After having quantified the phenomenon of reference rot for the three corpora, we sought to extrapolate our results in order to get an indication of the spread of reference rot in the broader scholarly communication system. Because reliable statistics exist pertaining to article publication patterns for STM subjects, we had purposely limited our corpora to those domains from the outset. We first set out to determine which of our corpora, if any, exhibited an evolution in the number of articles published per year that most closely resembles the evolution for the STM literature at large. If a corpus manifests such an evolution then our findings regarding reference rot for that corpus can be extrapolated to the STM literature by taking into account the ratio of the number of publications per year in that corpus to the total number of STM publications for that same year.

The report on Science and Engineering Indicators 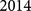 [40], in Table 5–20, provides an exact number of STM articles published globally for the years 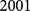 and 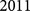: 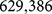 and 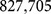, respectively (bold numbers in the “STM articles” row of Table 7). It also reports an annual average growth rate of 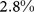 between those years. We used this growth rate to estimate the number of articles published in the years between 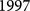 and 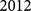 for which no exact figures are provided in the report (numbers not in bold in the “STM articles” row of Table 7). Our choice of this rate is supported by the 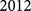 STM report [52], which states that the number of STM articles published each year has grown steadily by about 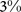 over the last two centuries.

**Table 7:** Reference rot: Extrapolated fraction of immune, healthy, and infected STM articles.

	1997	1998	1999	2000	2001	2002	2003	2004
Elsevier articles	39,480	41,110	39,027	38,494	39,314	37,232	35,971	37,439
Elsevier URI ratio	0.4%	0.7%	1.0%	2.1%	3.9%	7.3%	10.4%	13.2%
Elsevier RR ratio	0.4%	0.7%	0.9%	1.6%	3.0%	5.7%	7.2%	8.9%
STM articles	561,801	577,984	594,634	611,763	**629,386**	647,009	665,125	683,749
STM URI articles	2,247	4,046	5,946	12,847	24,546	47,232	69,173	90,255
STM RR articles	2,247	4,046	5,352	9,788	18,882	36,880	47,889	60,854

	2005	2006	2007	2008	2009	2010	2011	2012
Elsevier articles	35,735	39,236	41,065	42,032	44,162	44,716	49,150	50,877
Elsevier URI ratio	16.3%	18.6%	20.9%	23.3%	24.6%	25.2%	26.1%	26.6%
Elsevier RR Ratio	11.3%	12.7%	14.7%	17.9%	20.0%	20.9%	20.0%	21.0%
STM articles	702,894	722,575	742,807	763,605	784,986	806,966	**827,705**	852,789
STM URI articles	114,572	134,399	155,247	177,920	193,107	203,355	216,031	226,842
STM RR articles	79,427	91,767	109,193	136,685	156,997	168,656	165,541	179,085

**Figure 19:**
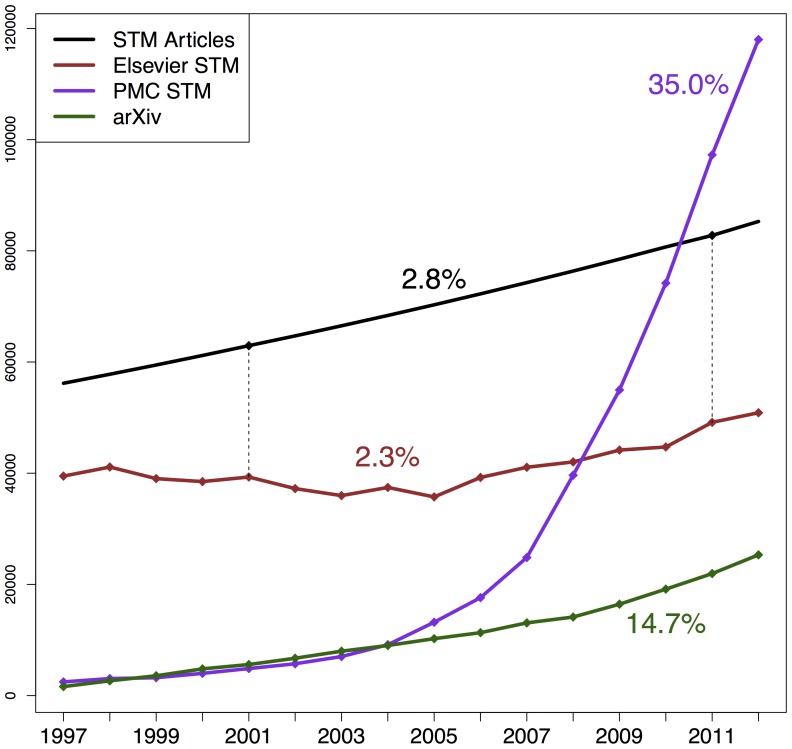
Growth rate of STM articles per publication year.

Fig. 19 shows the growth for STM article publications over time using the chosen average annual growth rate of 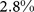 as a solid black line. Note that, in order to make the figure expressive, yet limit its size, we divided the number of articles per year for this line by 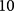. The data points provided for 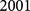 and 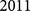 by [40] are indicated with a mark on the line. The same figure also shows the exact number of publications per year for each of our three corpora: the brown line for Elsevier, the purple line for PMC, and the green line for arXiv. In addition, each of these lines is labeled with its annual average growth rate. We observe that the evolution of our Elsevier corpus, which exhibits an annual average growth rate of 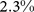, most closely resembles that of the STM article literature. The rates for arXiv and PMC are significantly higher for reasons touched upon in Section **Articles and URI References** and hence are less typical of the overall growth of STM literature. Supported by the fact that the Elsevier corpus was generated using a randomization, we decide that it forms an acceptable basis for an extrapolation attempt. Since the growth rate of our Elsevier corpus is lower than that of the STM literature in general, our extrapolation should be conservative rather than exaggerated.

**Figure 20:**
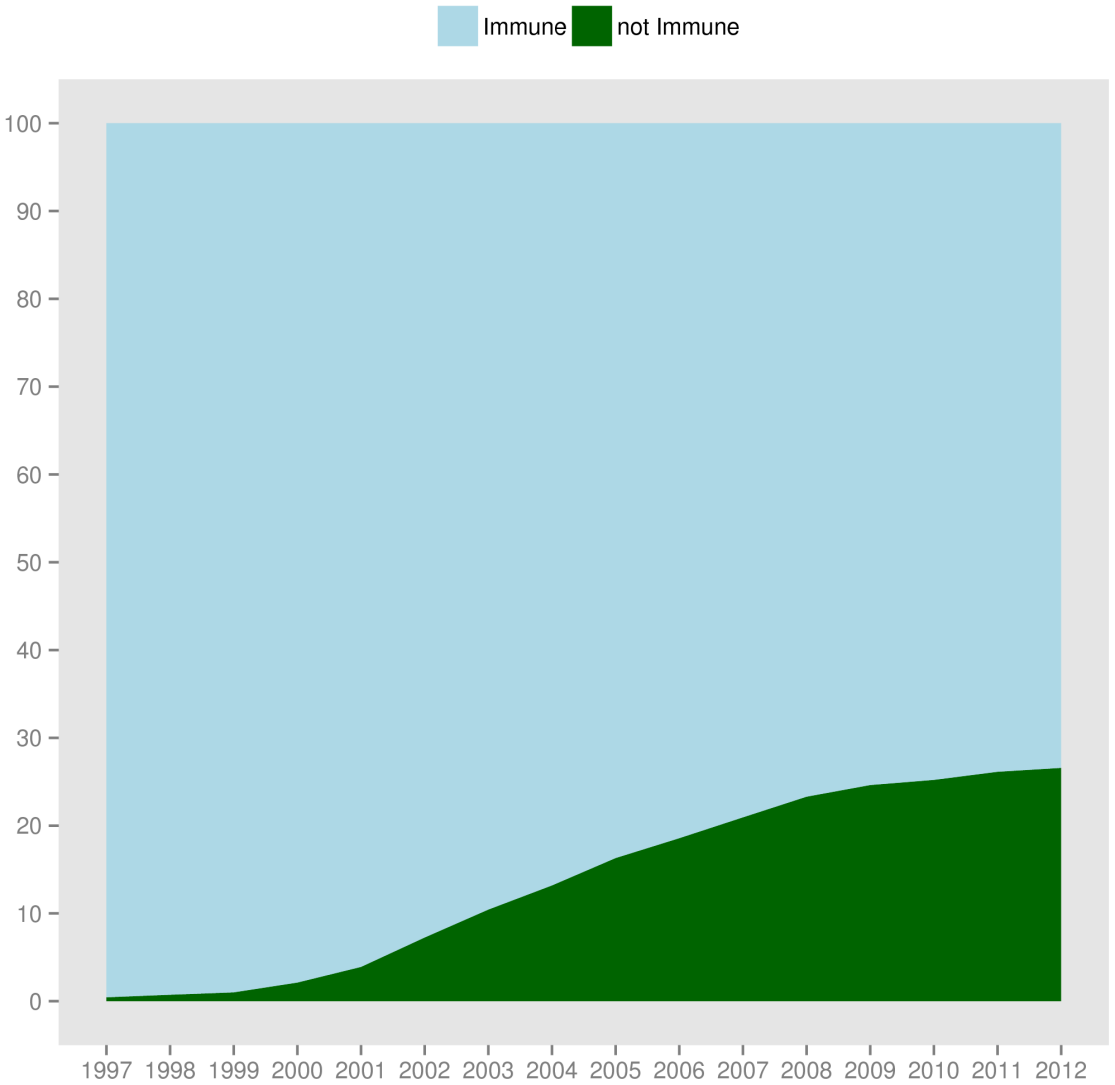
STM literature: extrapolated fraction of immune and not immune articles.

**Figure 21:**
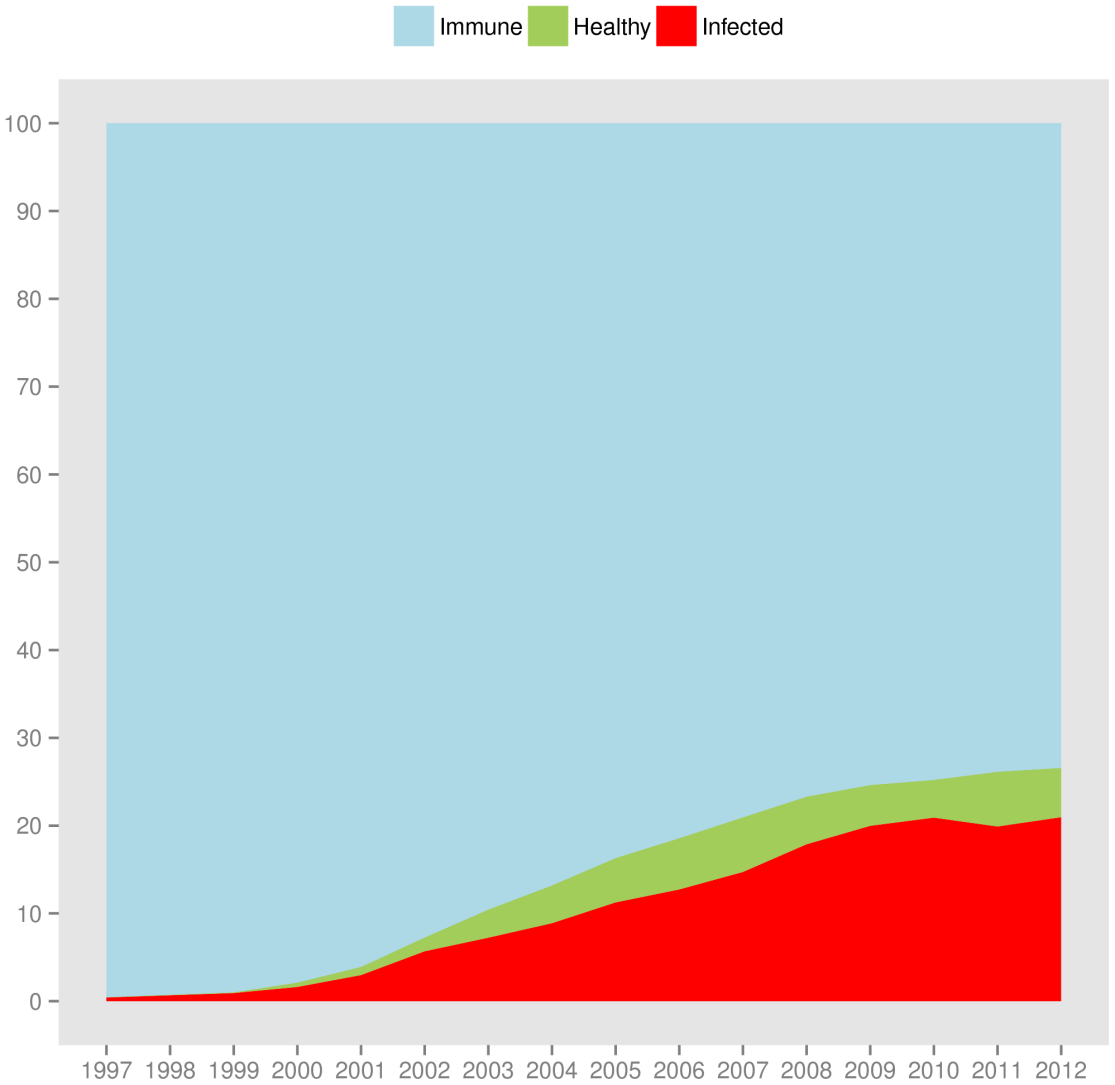
STM literature: extrapolated fraction of immune, healthy, and infected articles.

The first row of Table 7 (Elsevier articles) lists the number of articles in the Elsevier corpus between 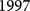 and 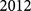, as previously reported in Table 5. The second row (Elsevier URI ratio) shows the percentage of Elsevier articles with URI references to web at large resources, whereas the third row (Elsevier RR ratio) shows the percentage of Elsevier articles that are infected with reference rot. The fourth row (STM articles) then provides the number of STM articles obtained as described above. The fifth (STM URI articles) and sixth row (STM RR articles) show extrapolated data, respectively, the extrapolated number of STM articles with URI references obtained using the ratios of the second row, and the extrapolated number of STM articles infected by reference rot obtained using the ratios of the third row. The results of this extrapolation suggests that, for recent publication years (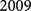
–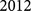), about one out of five STM articles is infected by reference rot. The number decreases for older publication years, consistent with earlier observations that a much higher percentage of older articles is immune. Fig. 20 visualizes the immune and not immune articles for our STM extrapolation. We observe that the fraction of immune articles (light blue area at the top of the figure) dominates for the early publication years but steadily shrinks over time, the direct result of articles increasingly referencing web at large resources and hence becoming more vulnerable to reference rot (dark green area). Fig. 21 adds a layer representing the infected fraction of STM articles (red area) to the plot. The figure shows that the growing number of articles that contain URI references (not immune) and that potentially could be healthy (light green area) is unfortunately to a very large extent infected by reference rot (red area).

Overall, our research indicates that:

- The fraction of STM articles that contain references to web at large resources steadily grows between 1997 and 2012. In 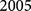, for example, the ratio was just over 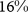 and it exceeded 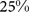 in 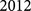.
- The vast majority of STM articles that contain references to web at large resources do suffer from reference rot. The infection rate between 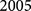 and 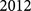 oscillates between 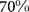 and 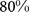.
- For the recent years, between 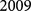 and 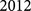, around 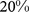 - one in five - of all STM articles suffer from reference rot.

To put this differently, for a large majority of STM articles published in the considered period, it is impossible the revisit the context - current and past - created by the web at large resources they reference.

## Discussion

Our research shows that, increasingly, articles reference web at large resources. It confirms a general finding of prior link rot studies, in our case derived from significantly vaster corpora, that links to such resources rot over time. As a result, many referenced resources can not be revisited some time after they were referenced. We also found that a significant amount of links remain operational. However, understanding that web resources are subject to content drift, following those links may eventually lead to content that is different than originally referenced. This consideration begs the question which is worse: following a link to a “404 Not Found” error message or to a page that may no longer be representative of the content that was originally referenced. A least the former is unambiguous, the latter is not.

Our research also found that web archive holdings, to a large extent, lack Mementos that provide sufficient guarantees of being representative of the originally referenced content. This should not come as a surprise as these Mementos are, from the perspective of web-based scholarly communication, the result of incidental archiving: they result from web archives conducting their regular crawling operations, and are not triggered by referencing activities in online scholarship. In order to improve on the status quo, a more pro-active approach towards archiving web at large resources that are being referenced should be considered.

Webcitation [53] was a pioneer in this problem domain when, years ago, it introduced the service that allows authors to archive, on demand, web resources they intend to reference. Meanwhile, other web archives including the Internet Archive, Perma.cc, and archive.today support such an on demand approach. But Webcitation has not been met with great success, possibly the result of a lack of authors' awareness regarding reference rot, possibly because the approach requires an explicit action by authors, likely because of both.

To a certain extent, portals like FigShare [54] and Zenodo [55] play in this problem domain as they allow authors to upload materials that might otherwise be posted to the web at large. The recent capability offered by these systems that allows creating a snapshot of a GitHub [56] repository, deposit it, and receive a DOI in return, serves as a good example. The main drivers for authors to do so is to contribute to open science and to receive a citable DOI, and, hence potentially credit for the contribution. But the net effect, from the perspective of the reference rot problem domain, is the creation of a snapshot of an otherwise evolving resource. Still, these services target materials created by authors, not, like web archives do, resources on the web irrespective of their authorship. Also, an open question remains to which extent such portals truly fulfill a long term archival function rather than being discovery and access environments.

In the solutions thread of Hiberlink, we explore pro-active archiving approaches intended to seamlessly integrate into the life cycle of an article and to require less explicit intervention by authors. One example is an experimental Zotero extension [57] that archives web resources as an author bookmarks them during note taking. Another is HiberActive, a service that can be integrated into the workflow of a repository or a manuscript submission system and that issues requests to web archives to archive all web at large resources referenced in submitted articles [58].

The solutions component of Hiberlink also explores how to best reference archived snapshots. The common and obvious approach, followed by Webcitation and Perma.cc, is to replace the original URI of the referenced resource with the URI of the Memento deposited in a web archive. This approach has several drawbacks. First, through removal of the original URI, it becomes impossible to revisit the originally referenced resource, for example, to determine what its content has become some time after referencing. Doing so can be rather relevant, for example, for software or dynamic scientific wiki pages. Second, the original URI is the key used to find Mementos of the resource in all web archives, using both their search interface and the Memento protocol. Removing the original URI is akin to throwing away that key: it makes it impossible to find Mementos in web archives other than the one in which the specific Memento was deposited. This means that the success of the approach is fully dependent on the long term existence of that one archive. If it permanently ceases to exist, for example, as a result of legal or financial pressure, or if it becomes temporally inoperative as a result of technical failure, the link to the Memento becomes rotten. Even worse, because the original URI was removed from the equation, it is impossible to use other web archives as a fallback mechanism. As such, in the approach that is currently common, one link rot problem is replaced by another.

This insight led Hiberlink to explore an alternative approach for referencing that consists of using the original URI for linking and annotating that link with archival information, namely, the URI of a specific snapshot of the referenced resource as intended by the reference and/or the datetime of referencing. When conveying this information in a machine-actionable manner, this referencing approach allows to revisit the referenced resource as it evolves by means of its original URI, to retrieve the specific Memento by means of its URI, and to find other temporally appropriate Mementos that may exist in various web archives through the combination of the original URI and the datetime of referencing.

Various alternatives for the syntax to express these link annotations are explored in the Missing Link document [59]. One approach leverages an extensibility mechanism built into HTML5 that allows annotating any link (HTML anchor element) with private attributes that have names starting with “data-”. Following this approach, the link <a href = "http://hiberlink.org"> can be turned into a reference that has increased temporal robustness <a href = "http://hiberlink.org" data-versionurl = http://archive.today/CT6mt data-versiondate = "2014-08-12">.

The Memento Time Travel extension for the Chrome browser [60] supports this approach and makes the original URI accessible through clicking, as usual, and the temporal attributes through right clicking. An HTML version of this paper's references is included in the Supporting Information section. All our references to web at large resources are annotated there in the manner described above and, hence, allow for web time travel of those links using Memento for Chrome.

Our research found that reference rot in scholarly communication is a significant problem that begs for the introduction of a robust solution. We researched and illustrated the problem by means of the STM journal literature but it is worth pointing out that other common outcomes of scholarship such as thesis and dissertations are not immune either. In addition, as the research process increasingly becomes web-based, a variety of research outcomes find their way to the web, many of them related and linked to other resources rather than being autonomous. Research objects [61], novel knowledge vessels that are increasingly used to communicate research results in computational sciences, form a good example as they are complex aggregations of interlinked and interdependent resources. Also, the reference rot problem is not constrained to the scholarly domain. Concerns regarding the temporal robustness of links have also been expressed with regard to Wikipedia articles [62], US Supreme Court decisions [63], government portals [64], Twitter [65], and blog platforms [66]. This suggests that an interoperable solution to the problem should be devised that can generally be adopted by various communities on the web, yet provides flexibility in implementation so that specific concerns of these communities can be met.

## Supporting Information

S1 Figure
**Mementos for URIs archived within** 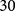 **days of being referenced - arXiv corpus.**
(TIF)

S2 Figure
**Mementos for URIs archived within** 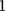 **day of being referenced - arXiv corpus.**
(TIF)

S3 Figure
**Mementos for URIs archived within** 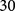 **days of being referenced - Elsevier corpus.**
(TIF)

S4 Figure
**Mementos for URIs archived within** 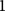 **day of being referenced - Elsevier corpus.**
(TIF)

S5 Figure
**Mementos for URIs archived within** 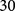 **days of being referenced - PMC corpus.**
(TIF)

S6 Figure
**Mementos for URIs archived within** 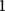 **day of being referenced - PMC corpus.**
(TIF)

S7 Figure
**URI references: corpora as sources, TLDs as targets - Mementos created within** 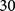 **days of referencing.**
(TIF)

S8 Figure
**URI references: corpora as sources, TLDs as targets - Mementos created within** 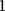 **day of referencing.**
(TIF)

S9 Figure **arXiv articles immune, healthy, and infected.**
(TIF)

S10 Figure **Elsevier articles immune, healthy, and infected.**
(TIF)

S11 Figure **PMC articles immune, healthy, and infected.**
(TIF)

S1 File **Annotated robust references to Web at Large resources.**
(HTML)
