# Supplementary figures and images for: Scholarly Context Not Found: One in Five Articles Suffers from Reference Rot
Source: PLoS One. 2014 Dec 26;9(12):e115253. doi: 10.1371/journal.pone.0115253 (PMC4277367; doi:10.1371/journal.pone.0115253)

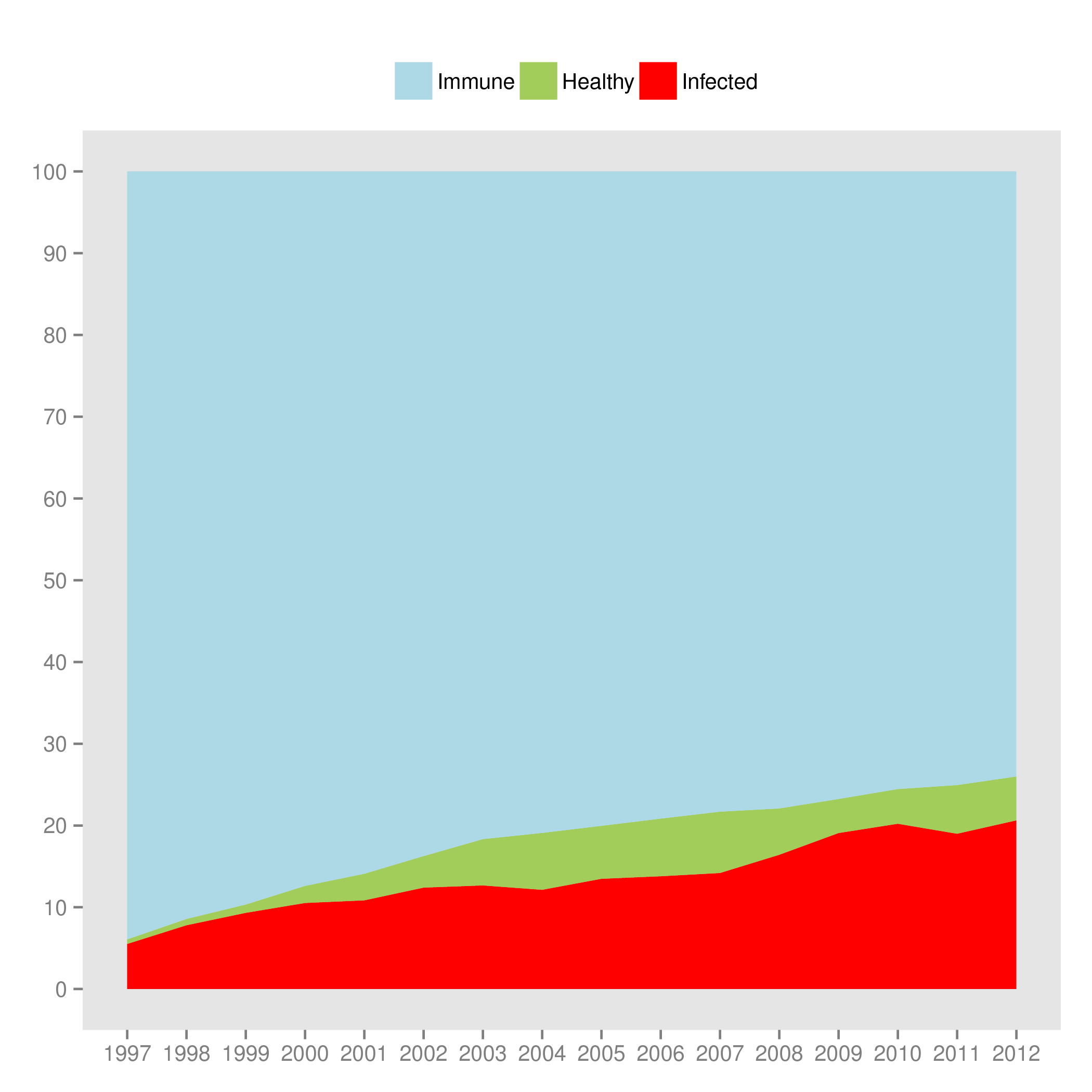

Supplement: S9 Figure — arXiv articles immune, healthy, and infected. (TIF) [file pone.0115253.s009.tif]

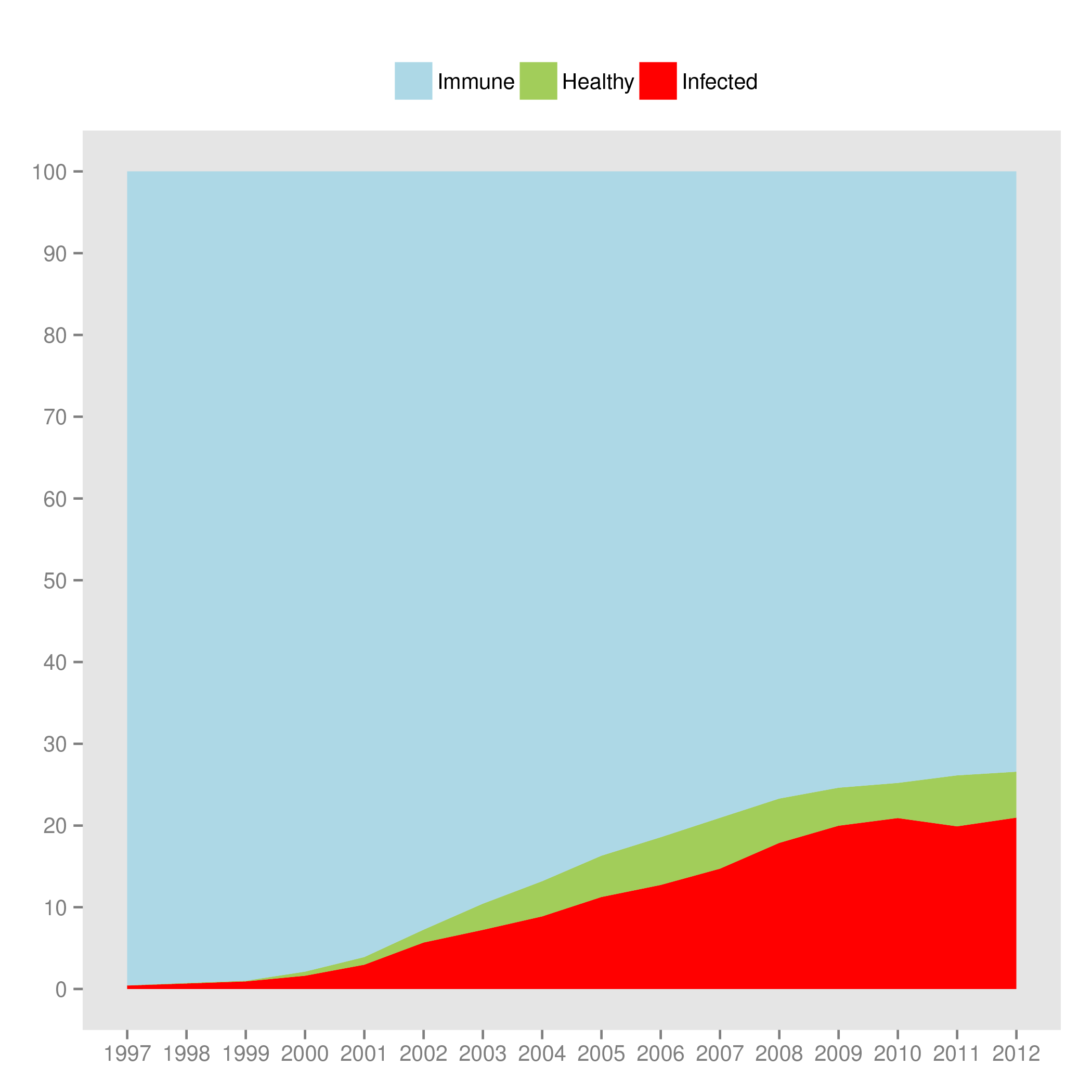

Supplement: S10 Figure — Elsevier articles immune, healthy, and infected. (TIF) [file pone.0115253.s010.tif]

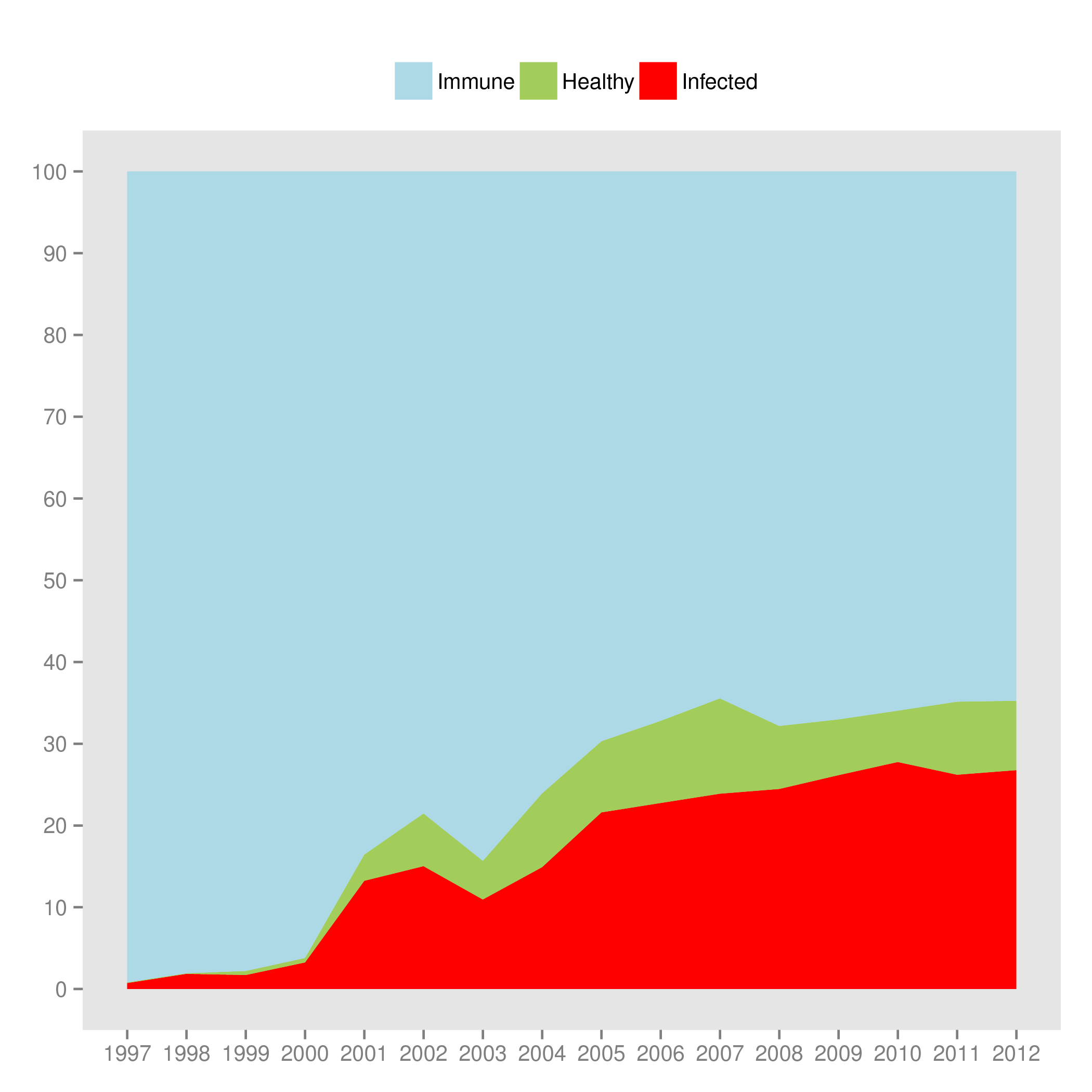

Supplement: S11 Figure — PMC articles immune, healthy, and infected. (TIF) [file pone.0115253.s011.tif]
