# Supplementary material for: Scholarly Context Not Found: One in Five Articles Suffers from Reference Rot
Source: PLoS One. 2014 Dec 26;9(12):e115253. doi: 10.1371/journal.pone.0115253 (PMC4277367; doi:10.1371/journal.pone.0115253)
Supplement: S1 File — Annotated robust references to Web at Large resources. (HTML) [file pone.0115253.s012.html]

Scholarly Context Not Found: One in Five Articles Suffers from Reference Rot


## Scholarly Context Not Found: One in Five Articles Suffers from Reference Rot

- Download the Memento for Chrome extension
- Right-click on links to utilize link annotation
- View the HTML source to see annotated, robust links

  
  

| Nr. | Reference |
| --- | --- |
| 1. | Hiberlink http://hiberlink.org/ Accessed: 1 Nov 2014 |
| 2. | Resolve a DOI Name http://dx.doi.org Accessed: 1 Nov 2014 |
| 3. | LOCKSS http://lockss.org/ Accessed: 1 Nov 2014 |
| 4. | CLOCKSS http://www.clockss.org/ Accessed: 1 Nov 2014 |
| 5. | Portico - A Digital Preservation and Electronic Archiving Service http://www.portico.org/ Accessed: 1 Nov 2014 |
| 6. | The Keepers Registry http://thekeepers.org/ Accessed: 1 Nov 2014 |
| 7. | Buckheit JB, Donoho DL (1995) Wavelab and reproducible research. In: Wavelets and Statistics, volume 103. pp. 55-81. |
| 8. | Berners-Lee T (1998). Cool URIs don't change http://www.w3.org/Provider/Style/URI.html Accessed: 26 Nov 2014 |
| 9. | Koehler WC (2002) Web Page Change and Persistence - A Four-Year Longitudinal Study. Journal of the American Society for Information Science and Technology 53: 162-171. |
| 10. | The Chesapeake Digital Preservation Group (2013). "Link Rot" and Legal Resources on the Web: A 2013 Analysis by the Chesapeake Digital Preservation Group. |
| 11. | Zittrain J, Albert K, Lessig L (2014) Perma: Scoping and addressing the problem of link and reference rot in legal citations. Harward Law Review Forum 127. |
| 12. | Wren JD (2004) 404 not found: the stability and persistence of urls published in medline. Bioinformatics 20: 668-672. |
| 13. | Wren JD (2008) Url decay in medlinea 4-year follow-up study. Bioinformatics 24: 1381-1385. |
| 14. | Duda JJ, Camp RJ (2008) Ecology in the information age: patterns of use and attrition rates of internet-based citations in esa journals, 1997-2005. Frontiers in Ecology and the Environment 6: 145-151. |
| 15. | Lawrence S, Pennock DM, Flake GW, Krovetz R, Coetzee FM, et al. (2001) Persistence of Web References in Scientific Research. Computer 34: 26-31. |
| 16. | Spinellis D (2003) The decay and failures of web references. Communications of the ACM 46: 71-77. |
| 17. | Dimitrova DV, Bugeja M (2007) The half-life of internet references cited in communication journals. New Media & Society 9: 811-826. |
| 18. | Aronsky D, Madani S, Carnevale RJ, Duda S, Feyder MT (2007) The prevalence and inaccessibility of internet references in the biomedical literature at the time of publication. Journal of the American Medical Informatics Association 14: 232-234. |
| 19. | Dellavalle RP, Hester EJ, Heilig LF, Drake AL, Kuntzman JW, et al. (2003) Information Science: Going, Going, Gone: Lost Internet References. Science 302: 787-788. |
| 20. | Hennessey J, Xijin Ge S (2013) A cross disciplinary study of link decay and the effectiveness of mitigation techniques. BMC Bioinformatics 14. |
| 21. | Sadat-Moosavi A, Isfandyari-Moghaddam A, Tajeddini O (2012) Accessibility of online resources cited in scholarly LIS journals: A study of emerald ISI-ranked journals. Aslib Proceedings 64: 178-192. |
| 22. | Dimitrova DV, Bugeja M (2007) Raising the dead: Recovery of decayed online citations. American Communication Journal 9. |
| 23. | Wagner C, Gebremichael MD, Taylor MK, Soltys MJ (2009) Disappearing act: decay of uniform resource locators in health care management journals. Journal of the Medical Library Association JMLA 97: 122-130. |
| 24. | Sanderson R, Phillips M, Van de Sompel H (2011) Analyzing the Persistence of Referenced Web Resources with Memento. Technical Report arXiv:1105.3459. |
| 25. | Brewington B, Cybenko G (2000) Keeping Up With the Changing Web. Computer 33: 52-58. |
| 26. | Cho J, Garcia-Molina H (2000) The Evolution of the Web and Implications for an Incremental Crawler. In: Proceedings of VLDB '00. pp. 200-209. |
| 27. | Fetterly D, Manasse M, Najork M, Wiener J (2003) A Large-Scale Study of the Evolution of Web Pages. In: Proceedings of WWW '03. pp. 669-678. |
| 28. | Ntoulas A, Cho J, Olston C (2004) What's New on the Web?: The Evolution of the Web from a Search Engine Perspective. In: Proceedings of WWW '04. pp. 1-12. |
| 29. | Adar E, Teevan J, Dumais ST, Elsas JL (2009) The Web Changes Everything: Understanding the Dynamics of Web Content. In: Proceedings of WSDM '09. pp. 282-291. |
| 30. | Adar E, Dontcheva M, Fogarty J, Weld DS (2008) Zoetrope: Interacting with the Ephemeral Web. In: Proceedings of UIST '08. pp. 239-248. |
| 31. | Klein M, Nelson ML (2014) Moved but not gone: an evaluation of real-time methods for discovering replacement web pages. International Journal on Digital Libraries 14: 17-38. |
| 32. | Klein M, Nelson ML (2008) Revisiting Lexical Signatures to (Re-)Discover Web Pages. In: Proceedings of ECDL '08. pp. 371'382. |
| 33. | Van de Sompel H, Nelson ML, Sanderson R (2013). RFC 7089: HTTP Framework for Time-Based Access to Resource States - Memento. |
| 34. | arXiv.org e-Print archive http://arxiv.org/ Accessed: 1 Nov 2014 |
| 35. | Elsevier http://www.elsevier.com/ Accessed: 1 Nov 2014 |
| 36. | Randoim http://labs.crossref.org/randoim/ Accessed: 1 Nov 2014 |
| 37. | CrossRef Text and Data Mining http://tdmsupport.crossref.org/ Accessed: 1 Nov 2014 |
| 38. | PubMed Central http://www.ncbi.nlm.nih.gov/pmc/ Accessed: 1 Nov 2014 |
| 39. | Ulrich's knowledgebase http://www.proquest.com/products-services/Ulrichsweb.html Accessed: 1 Nov 2014 |
| 41. | PDFTOHTML http://pdftohtml.sourceforge.net/ Accessed: 1 Nov 2014 |
| 42. | Zhou K, Tobin R, Grover C (2014) Large-Scale Extraction and Analysis of Web Link References in Scholarly Articles. In: Proceedings of DL '14 (to appear). |
| 43. | Reverse domain lookup http://labs.crossref.org/reverse-domain-lookup/ Accessed: 1 Nov 2014 |
| 44. | IANA http://www.icann.org/resources/pages/tlds-2012-02-25-en Accessed: 1 Nov 2014 |
| 45. | Cheshire S, Krochmal M (2013). RFC 6761: Special-Use Domain Names. |
| 46. | AlSum A, Weigle MC, Nelson ML, Van de Sompel H (2014) Profiling web archive coverage for top-level domain and content language. International Journal on Digital Libraries 14: 149-166. |
| 47. | NIH open access mandate made permanent http://sciencecommons.org/weblog/archives/2009/03/17/nih-mandate-made-permanent/ Accessed: 1 Nov 2014 |
| 48. | NIHMS statistics http://www.nihms.nih.gov/stats/index.shtml Accessed: 1 Nov 2014 |
| 49. | DOI display guidelines http://http://www.crossref.org/02publishers/doi\_display\_guidelines.html Accessed: 26 Nov 2014 |
| 50. | Douglis F, Feldmann A, Krishnamurthy B, Mogul JC (1997) Rate of change and other metrics: a live study of the world wide web. In: USENIX Symposium on Internet Technologies and Systems. volume 119. |
| 51. | Tyler DC, McNeil B (2003) Librarians and link rot: A comparative analysis with some methodological considerations. portal: Libraries and the Academy 3: 615-632. |
| 52. | Ware M, Mabe M (2012). The STM report. An overview of scientific and scholarly journal publishing. |
| 53. | WebCite http://webcitation.org/ Accessed: 1 Nov 2014 |
| 54. | Figshare http://figshare.com/ Accessed: 1 Nov 2014 |
| 55. | ZENODO http://zenodo.org/ Accessed: 1 Nov 2014 |
| 56. | GitHub https://github.com/ Accessed: 1 Nov 2014 |
| 57. | Zotero extension https://github.com/rwincewicz/zotero\_hiberlink Accessed: 1 Nov 2014 |
| 58. | Van de Sompel H, Klein M, Shankar H (2014) Towards robust hyperlinks for web-based scholarly communication. In: Intelligent Computer Mathematics, volume 8543. pp. 12-25. |
| 59. | Thoughts on Referencing, Linking, Reference Rot http://mementoweb.org/missing-link/ Accessed: 1 Nov 2014 |
| 60. | Memento Time Travel https://chrome.google.com/webstore/detail/memento-time-travel/jgbfpjledahoajcppakbgilmojkaghgm?hl=en≷=US Accessed: 1 Nov 2014 |
| 61. | Bechhofer S, De Roure D, Gamble M, Goble C, Buchan I (2010) Research objects: Towards exchange and reuse of digital knowledge. The Future of the Web for Collaborative Science |
| 62. | Wikipedia talk: Link rot http://en.wikipedia.org/wiki/Wikipedia\_talk:Link\_rot Accessed: 1 Nov 2014 |
| 63. | In supreme court opinions, web links to nowhere http://www.nytimes.com/2013/09/24/us/politics/in-supreme-court-opinions-clicks-that-lead-nowhere.html Accessed: 1 Nov 2014 |
| 64. | The National Archives http://nationalarchives.gov.uk/webarchive/webmasters.htm Accessed: 1 Nov 2014 |
| 65. | SalahEldeen H, Nelson ML (2012) Losing My Revolution: How Many Resources Shared on Social Media Have Been Lost? In: Proceedings of TPDL '12. pp. 125-137. |
| 66. | Broken wordpress links http://inkdroid.org/journal/2013/10/28/broken-wordpress-links/ Accessed: 1 Nov 2014 |
